# Unprecedentedly high activity and selectivity for hydrogenation of nitroarenes with single atomic Co_1_-N_3_P_1_ sites

**DOI:** 10.1038/s41467-022-28367-9

**Published:** 2022-02-07

**Authors:** Hongqiang Jin, Peipei Li, Peixin Cui, Jinan Shi, Wu Zhou, Xiaohu Yu, Weiguo Song, Changyan Cao

**Affiliations:** 1 Beijing National Laboratory for Molecular Sciences, CAS Research/Education Center for Excellence in Molecular Sciences, Laboratory of Molecular Nanostructures and Nanotechnology, Institute of Chemistry, Chinese Academy of Sciences, Beijing, 100190 China; 2 School of Chemical Sciences, University of Chinese Academy of Sciences, Beijing, 100049 China; 3 Key Laboratory of Soil Environment and Pollution Remediation, Institute of Soil Science, Chinese Academy of Sciences, Nanjing, 210008 China; 4 School of Physical Sciences, CAS Key Laboratory of Vacuum Physics, University of Chinese Academy of Sciences, Beijing, 100049 China; 5 CAS Center for Excellence in Topological Quantum Computation, University of Chinese Academy of Sciences, Beijing, 100049 China; 6 Institute of Theoretical and Computational Chemistry, Shaanxi Key Laboratory of Catalysis, School of Chemical & Environment Sciences, Shaanxi University of Technology, Hanzhong, 723000 China

**Keywords:** Heterogeneous catalysis, Structural properties, Catalyst synthesis

## Abstract

Transition metal single atom catalysts (SACs) with M_1_-N_x_ coordination configuration have shown outstanding activity and selectivity for hydrogenation of nitroarenes. Modulating the atomic coordination structure has emerged as a promising strategy to further improve the catalytic performance. Herein, we report an atomic Co_1_/NPC catalyst with unsymmetrical single Co_1_-N_3_P_1_ sites that displays unprecedentedly high activity and chemoselectivity for hydrogenation of functionalized nitroarenes. Compared to the most popular Co_1_-N_4_ coordination, the electron density of Co atom in Co_1_-N_3_P_1_ is increased, which is more favorable for H_2_ dissociation as verified by kinetic isotope effect and density functional theory calculation results. In nitrobenzene hydrogenation reaction, the as-synthesized Co_1_-N_3_P_1_ SAC exhibits a turnover frequency of 6560 h^−1^, which is 60-fold higher than that of Co_1_-N_4_ SAC and one order of magnitude higher than the state-of-the-art M_1_-N_x_-C SACs in literatures. Furthermore, Co_1_-N_3_P_1_ SAC shows superior selectivity (>99%) toward many substituted nitroarenes with co-existence of other sensitive reducible groups. This work is an excellent example of relationship between catalytic performance and the coordination environment of SACs, and offers a potential practical catalyst for aromatic amine synthesis by hydrogenation of nitroarenes.

## Abstract (web summary)

Modulating the atomic coordination structure has emerged as a promising strategy to further improve catalytic performance. Here, the authors report an atomic Co1/NPC catalyst with unsymmetrical single Co1N3P1 sites that displays high activity and chemoselectivity for hydrogenation of functionalized nitroarenes.

## Introduction

Chemoselective hydrogenation of nitroarenes is a key reaction in the fine chemical industry and has wide applications in the synthesis of pigments and pharmaceuticals^1–4^. Noble metal nanocatalysts (e.g., Pt, Au, and Pd) are usually used for this reaction^5–7^. However, noble metal catalysts are costly and their high activities usually come with unsatisfactory selectivity against many substituted nitroarenes^8–11^. Since Beller et al. reported highly selective traditional metal catalysts based on Co_3_O_4_@N/C and Fe_3_O_4_@N/C, it has sparked intensive research interest in this type of catalysts^1,12^. Among them, transition metal single-atom catalysts (SACs) with M_1_-N_x_-C (M = Fe, Co, Ni, x = 2–6) coordination configuration exhibited much better activities than their counterpart nanoparticles while maintaining high selectivity, owing to their maximum atom efficiency and particular electronic structure^13–18^. Recently, Wang et al. found that the electron density of Ni single atoms increased with the decrease of Ni-N coordination numbers (CN), and the capability of Ni single sites to dissociate H_2_ was greatly enhanced, leading to higher catalytic activity in chemoselective hydrogenation of functionalized nitroarenes^19^. This result suggests that the catalytic activity of M_1_-N_x_-C can also be enhanced by adjusting the coordination structure of transition metal SACs.

In most SACs, the central metal atoms were stabilized by coordination bonds with N, S, O, etc. atoms within support matrix^20–29^. The electronic and geometric structures of central metal atoms can be adjusted by tailoring the coordination environment, which would change the absorption energy of reactants on metal atoms and thus influence the catalytic process^20,23,28,30^. For SACs with M_1_-N_x_-C sites, the symmetric electronic distribution may limit the activation of reactants, thereby leading to hampered catalytic kinetics and performances^20,31^. Recent studies have found that introducing heteroatom P for an unsymmetrical N/P mixed-coordination can further modulate the electronic properties of center metal atoms. The unsymmetrical geometric structure can evoke the distortion of electronic density and alter the d-band center^20,23,28,31,32^. For example, Yuan et al. prepared an N/P dual-coordinated Fe single-atom catalyst, which was more favorable for the adsorption of oxygen intermediates for ORR in fuel cell^23^. Li et al. reported that a Fe_1_-N_3_P_1_ single-atom nanozyme exhibited peroxidase-like catalytic activity, and the high activity was ascribed to the less positive charge on Fe atoms as P atoms are electron donors^32^. Thus, we anticipated that constructing the unsymmetrical N/P dual-coordinated transition metal SACs would improve the catalytic performance for the hydrogenation of nitroarenes.

In this work, we report an N/P dual-coordinated Co SAC (denoted as Co_1_/NPC) with Co_1_-N_3_P_1_ coordination structure and investigate its catalytic performance for chemoselective hydrogenation of nitroarenes. The single atomic feature and coordination structure of the Co_1_-N_3_P_1_ site are characterized through aberration-corrected high angle annular dark-field scanning transmission electron microscopy (AC HAADF-STEM), atomic-resolution electron energy-loss spectroscopy (EELS), X-ray photoelectron spectroscopy (XPS), and X-ray absorption spectrum (XAS). In nitrobenzene hydrogenation reaction, the Co_1_-N_3_P_1_ SAC exhibits a turnover frequency of 6560 h^−1^, which is 60 times higher than that of Co_1_-N_4_ SAC and 10 times higher than the state-of-the-art M_1_-N_x_-C SACs in literatures. Furthermore, Co_1_-N_3_P_1_ SAC shows superior selectivity (>99%) toward many substituted nitroarenes with the co-existence of other sensitive reducible groups. The unprecedentedly high activity of Co_1_-N_3_P_1_ SAC can be ascribed to the upshift d-band center of Co single atoms, which is more favorable for H_2_ dissociation as verified by the kinetic isotope effect and density functional theory calculation results. This is an excellent example of such an unsymmetrical N/P dual-coordinated structure of metal SACs in hydrogenation.

## Results

### Structural characterization

**Fig. 1:**
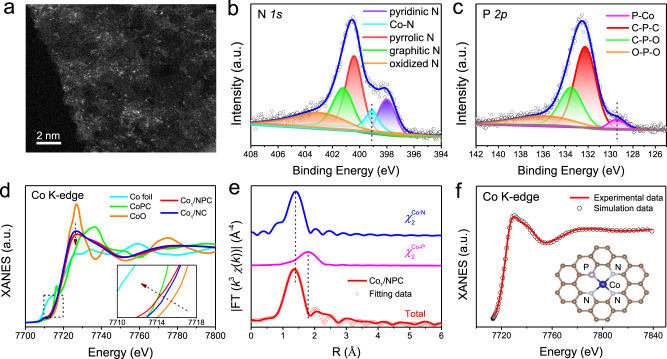
Structural Characterizations of Co_1_/NPC. **a** AC HAADF-STEM image of Co_1_/NPC. **b** N 1*s* and **c** P 2*p* XPS spectra of Co_1_/NPC. **d** Co K-edge XANES spectra. **e** k^3^-weight FT-EXAFS fitting curves of Co_1_/NPC. Curves from top to bottom are the Co-N, Co-P backscattering pathways and fitting total signal (red circle) superimposed on the experimental signal (red line). **f** The experimental XANES curve in comparison with the calculated XANES data of Co_1_-N_3_P_1_ site in Co_1_/NPC sample. Inset: the schematic atomic structure of Co_1_/NPC derived from the EXAFS results.

Supplementary Fig. 1 illustrates the synthesis procedures for preparing N/P dual-coordinated Co SAC (denoted as Co_1_/NPC) via a two-step process. First, tannic acid, (2-Aminoethyl)phosphonic acid (AePA), and cobalt ion precursors were co-adsorbed on the surface of graphitic carbon nitride (g-C_3_N_4_) nanosheets; then the resultant powder was subjected to pyrolysis under flowing Ar gas at 900 °C to obtain Co_1_/NPC, where the AePA was absent and the introduced-P species served as the donors for anchoring Co atoms (Supplementary Fig. 2). For comparison, N-coordinated Co SAC (denoted as Co_1_/NC) was also prepared via the same procedure without the addition of AePA. As exhibited in Raman spectra, the carbon matrices in both Co_1_/NPC and Co_1_/NC were disordered with a large number of defects (Supplementary Fig. 3). Only two broad peaks at ~24.3° and 42.6° could be observed from their X-ray diffraction (XRD) patterns (Supplementary Fig. 4), corresponding to (002) and (101) planes of carbon, suggesting highly dispersed states of Co species in both of two samples. Further increasing the pyrolysis temperature of Co_1_/NPC to 1000 °C led to the formation of Co_2_P nanoparticles (Supplementary Figs. 4, 5). Scanning electron microscopy (SEM) and transmission electron microscopy (TEM) images show that both catalysts retain a two-dimensional layered structure and no obvious nanoparticles are observed (Supplementary Figs. 6, 7). Energy-dispersive spectroscopy (EDS) mappings reveal Co elements are distributed uniformly over the entire samples (Supplementary Figs. 8, 9). Additionally, Co single-atom feature in Co_1_/NPC and Co_1_/NC is directly observed by AC HAADF-STEM, as reflected by the highly dispersed bright dots due to the heavy Z-contrast (Fig. 1a and Supplementary Fig. 10). The Co loading in Co_1_/NPC was ~0.45 wt% as determined by the inductively coupled plasma mass spectroscopy (ICP-MS) analysis (Supplementary Table 1). The porosity features of Co_1_/NC and Co_1_/NPC were investigated using nitrogen physisorption measurements. Both catalysts displayed characteristics of IV-type N_2_ adsorption-desorption isotherms, suggesting the existence of mesopores, which would be beneficial for the exposure of active sites and mass transportation (Supplementary Fig. 11). The calculated BET-specific surface area of Co_1_/NC and Co_1_/NPC were 512 and 471 m^2^ g^−1^, respectively.

XPS was then applied to reveal the chemical structures of both Co SACs. In N 1*s* spectra, besides pyridinic N, pyrrolic N, graphitic N, and oxidized N species, a peak at 399.1 eV corresponding to Co-N can be distinguished^33^, indicating the existence of N coordination environment with Co single atoms in both catalysts (Fig. 1b and Supplementary Fig. 12). Note that from P 2*p* spectra in Fig. 1c, an obvious peak at ~129.3 eV corresponding to Co-P bond was presented in Co_1_/NPC^23,31^, which can also be observed in the comparison Co_2_P NPs/C (Supplementary Fig. 13); while it is absent in Co_1_/NC sample. These results suggest that the atomically dispersed Co atoms possess N/P dual-coordinated configuration in Co_1_/NPC, while only N-coordinated configuration in Co_1_/NC.

To further determine the coordination environment of Co single atoms, X-ray absorption fine structure (XAFS) measurements were conducted. Figure 1d shows the Co K-edge X-ray absorption near-edge structure (XANES) curves of Co_1_/NC and Co_1_/NPC, with Co foil, CoO, and cobalt phthalocyanine (CoPc) as reference samples. It can be seen that the absorption threshold positions for Co_1_/NC and Co_1_/NPC are located between Co foil and CoO, suggesting that the valence states of Co species are between 0 and +2 in both two catalysts. Moreover, the Co K-edge position and white line of Co_1_/NPC are lower than that of Co_1_/NC (inset of Fig. 1d), which indicates that Co atoms in Co_1_/NPC possess more negative charges than Co_1_/NC. Such difference could be attributed to the less electron transfer from Co to P because of the weaker electronegativity of P than N atoms^20^. The Fourier-transformed *k*^3^-weighted EXAFS (FT-EXAFS) spectra demonstrated that both Co_1_/NC and Co_1_/NPC only exhibited a prominent peak at 1.38 Å (without phase shift), no Co-Co peaks at 2.17 Å or larger bond distances were detected, confirming atomically dispersed Co species in Co_1_/NC and Co_1_/NPC (Supplementary Figs. 14, 15). The coordination configuration of Co moieties was further surveyed using quantitative least-squares EXAFS curve-fitting. The EXAFS spectrum of Co_1_/NPC was investigated by utilizing Co-N and Co-P backscattering pathways. The best-fitting analysis displays that the main peak at 1.38 Å could be satisfactorily interpreted as Co-N first-shell coordination with CN = 3.2 ± 0.1 and the shoulder peak at 1.77 Å originated from Co-P contribution with CN = 0.9 ± 0.1 (Fig. 1e and Supplementary Table 2), suggesting the possible Co_1_-N_3_P_1_ configuration in Co_1_/NPC. For comparison, fitting of Co_1_/NC resulted in an average of about four N atoms with a distance of 1.37 Å (Supplementary Fig. 14 and Supplementary Table 2). In order to better confirm the proposed configurations, the theoretical XANES spectrum were simulated based on the Co_1_-N_3_P_1_ model as well as Co_1_-N_4_, which presented a good agreement with the experimental data, indicating the rationality of the two structures (Fig. 1f and Supplementary Fig. 16).

**Fig. 2:**
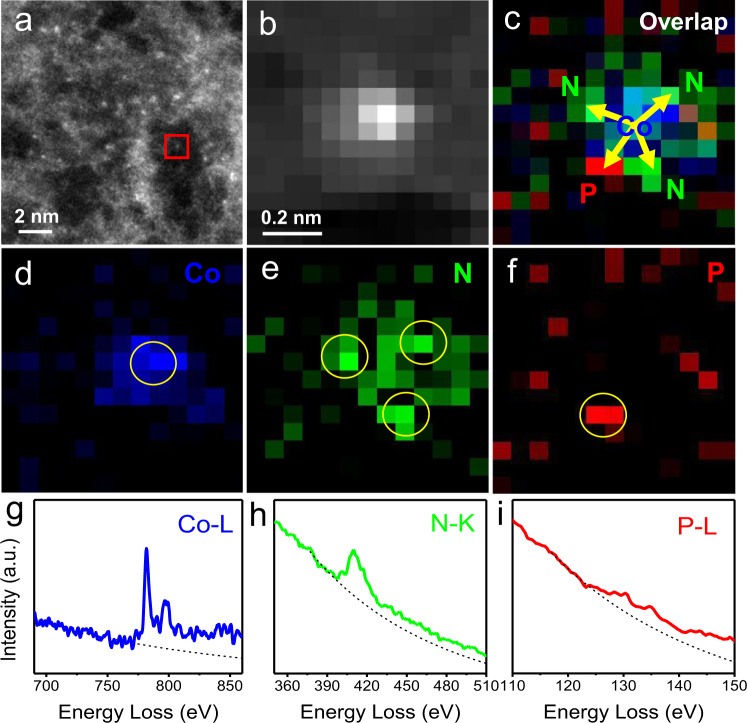
Atomic-resolution EELS analysis of Co_1_/NPC sample. **a** AC HAADF-STEM image of Co_1_/NPC sample at a relatively low beam current. **b** HAADF image acquired simultaneously with atomic-resolution EELS mapping of Co_1_/NPC at the red square area in (**a**). **c** The overlap map of P, N, and Co elements. **d**–**f** Elemental maps of Co, N, P, respectively. **g**–**i** The EELS spectra extracted at the yellow circled position from (**d**–**f**), respectively.

Besides, we performed atomic-resolution electron energy-loss spectroscopy (EELS) analysis at a relatively low beam current to minimize electron-beam perturbations to provide strong evidence of Co_1_-N_3_P_1_ structure (Fig. 2a, b). The extracted Co L-edge EELS spectrum from Fig. 2d presents a clear Co signal (Fig. 2g), providing direct evidence for the presence of atomically dispersed Co species. More importantly, the existence of N, P dual-coordination vicinal to Co site is revealed by identifying the surrounding heteroatoms. From the N, P, and overlap maps (Fig. 2e, f, c), three N atoms (green) and one P atom (red) exist around the Co site. Atomic-scale N K-edge and P L-edge EELS spectra collected at the corresponding positions from Fig. 2e, f are further demonstrated by the N and P signals (Fig. 2h, i). This forcefully confirms the Co_1_-N_3_P_1_ configuration in Co_1_/NPC sample. Moreover, the formation energy of the Co_1_-N_3_P_1_ structure in the Co_1_/NPC sample was estimated to be about −0.864 eV by DFT calculation, indicating the high stability of the proposed configuration (Supplementary Fig. 17). These results revealed that the Co single sites in Co_1_/NPC were stabilized with N/P dual-coordinated structure, forming an unsymmetrical Co_1_-N_3_P_1_ geometric configuration (as depicted in Fig. 1f), which is different from the Co site in Co_1_/NC with traditional in-plane Co_1_-N_4_ configuration.

### Catalytic performance

**Fig. 3:**
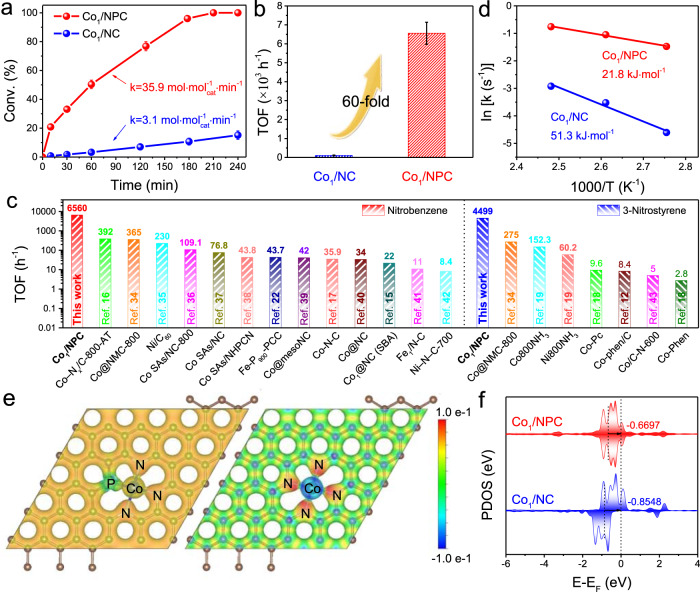
Catalytic performances and kinetic process studies. **a** Time course of nitrobenzene conversions over Co_1_/NPC and Co_1_/NC samples. The standard deviations were derived from three independent trials. Reaction conditions: 5 mg catalyst, 2 mmol nitrobenzene, 40 mL EtOH/H_2_O (v:v = 4:1), 110 °C, 3 MPa H_2_. **b** TOF of Co_1_/NPC and Co_1_/NC for the nitrobenzene hydrogenation. The TOF values were calculated at about 20% conversion. **c** The TOF values comparison of recently reported transition metal catalysts. **d** The experimental Arrhenius plots of Co_1_/NPC and Co_1_/NC. **e** Electron-density isosurface of Co atoms in two models. Blue color indicates positive charges and red color indicates negative charge. **f** Partial density of states (PDOS) of Co atoms in Co_1_/NPC and Co_1_/NC, the zero-energy corresponds to the Fermi level, and the d-band centers are inserted with the short dot.

To evaluate the catalytic performances of the as-prepared Co SACs for the hydrogenation of nitroarenes, nitrobenzene is first chosen as a probe molecule. The reaction kinetics with Co_1_/NC and Co_1_/NPC were obtained at 110 °C with 3 MPa H_2_ in a Teflon-lined stainless steel autoclave. As shown in Fig. 3a, Co_1_/NPC exhibits significantly higher activity than that of Co_1_/NC. Nitrobenzene was completely converted with >99% amine selectivity in 210 min with Co_1_/NPC, while less than 20% conversion was observed with Co_1_/NC under the same reaction condition. In addition, no conversion was observed with NC and NPC supports, suggesting that atomic Co site was active species in both Co_1_/NC and Co_1_/NPC catalysts (Supplementary Fig. 18). The reaction rate (k) for hydrogenation of nitrobenzene over Co_1_/NPC could reach as high as 35.9 mol mol^−1^ min^−1^, which is ten times higher than that with Co_1_/NC (3.1 mol mol^−1^ min^−1^).

The turnover frequency value (TOF) (based on the substrate conversion at about 20%) of Co_1_/NPC is calculated to be 6560 h^−1^, which is over 60 times higher than that with Co_1_/NC (108 h^−1^) (Fig. 3b). Besides nitrobenzene, Co_1_/NPC also exhibited superior high activity and excellent selectivity (>99.7%) for hydrogenation of 3-nitrostyrene with TOF of 4499 h^−1^. The impressive activity of Co_1_/NPC is ten times higher than the state-of-the-art M_1_-N_x_-C SACs in literature (Fig. 3c and Supplementary Table 3)^34–43^. Such excellent catalytic performance of Co_1_/NPC sample inspired us to carry out the reaction under milder conditions (e.g., 40 °C, 1 bar H_2_). A high nitrobenzene conversion of 97.2% was achieved within 5 h (Supplementary Fig. 19). To further compare the catalytic performance between Co_1_/NC and Co_1_/NPC, the apparent activation energies of these two catalysts were measured (Supplementary Fig. 20 and Supplementary Table 4). As shown in Fig. 3d, the calculated activation energy of Co_1_/NPC catalyst is about 21.8 kJ mol^−1^, which is much lower than that of Co_1_/NC (51.3 kJ mol^−1^).

In order to clarify the intrinsic higher activity of Co_1_-N_3_P_1_, the electronic properties of the central metal sites over Co_1_/NC and Co_1_/NPC are examined by electron-density isosurface and partial density of states from DFT calculations. Different charge distributions of the two models are observed (Fig. 3e). Compare to the Co_1_-N_4_ configuration, the symmetric electron structure is broken by introducing heteroatom P in Co_1_-N_3_P_1_. The Bader charge of the Co_1_-N_3_P_1_ site is estimated to be +0.81 *e*, while the Co_1_-N_4_ site is +0.97 *e*, indicating the Co atom in the Co_1_-N_3_P_1_ site carries more charge since the P element in the Co_1_/NPC transfers 2.308 *e* to support, which is consistent with the XAFS results. Moreover, the Co d-band center of Co_1_/NPC is up-shifted, much closer to the Fermi level (Fig. 3f). As a result, the antibonding state of Co atoms and adsorbed H_2_ species are more occupied, then such change enhances the capabilities of H_2_ dissociation^6,44^. Thus, Co_1_/NPC catalyst with Co_1_-N_3_P_1_ configuration exhibits much higher activity than that of Co_1_/NC with Co_1_-N_4_ configuration.

### Catalytic hydrogenation mechanism

**Fig. 4:**
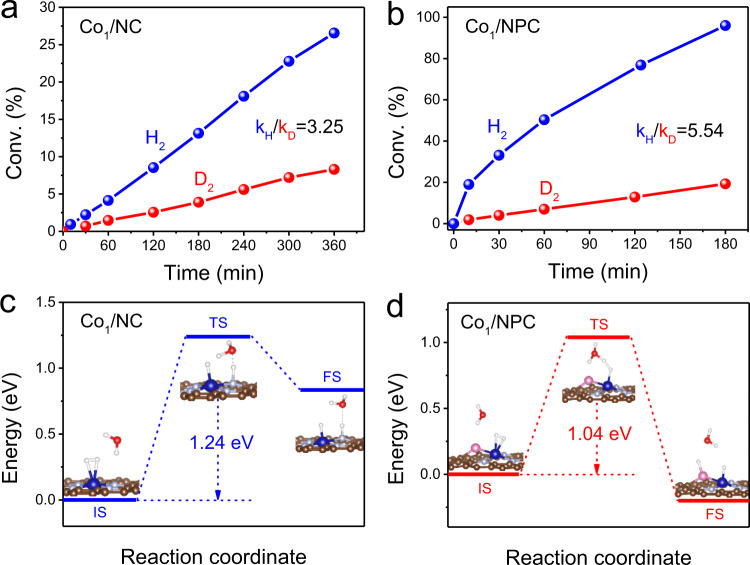
Catalytic mechanism for the hydrogenation of nitrobenzene. Primary isotope effect observed on **a** Co_1_/NC and **b** Co_1_/NPC. Reaction conditions: 5 mg catalyst, 2 mmol nitrobenzene, 40 mL EtOH/H_2_O (D_2_O) (v:v = 4:1), 110 °C, 3 MPa H_2_ (D_2_). Energies profiles for H_2_ dissociation pathways of **c** Co_1_/NC and **d** Co_1_/NPC samples. IS initial state, TS transition state, FS final state.

Such a large activity difference between Co_1_/NC and Co_1_/NPC implies that the hydrogenation activity is closely correlated with local coordination structure, which influences the electronic structure of Co single atoms. In order to elucidate the reaction mechanisms on both catalysts, we carried out a kinetic isotope effect (KIE) study to examine the H_2_ dissociation step. Using D_2_ for nitrobenzene hydrogenation, the reaction rate was slowed down by a factor of 3.25 for Co_1_/NC (Fig. 4a). For comparison, a larger KIE (k_H_/k_D_ = 5.54) was observed on Co_1_/NPC catalyst (Fig. 4b). These results suggest that H_2_ dissociation undergoes heterolytic cleavage on both Co_1_/NC and Co_1_/NPC^45–47^.

It is generally accepted that heterolytic cleavage of H_2_ on metal single atoms occurs to form metal-H^δ-^ and heteroatom-H^δ+^ ^45,48^. Therefore, DFT calculations were further performed to understand the H_2_ heterolytic cleavage on Co_1_/NC and Co_1_/NPC, respectively. As shown in Supplementary Fig. 21, the H_2_ molecule is adsorbed on the Co atom of Co_1_/NPC with adsorption energy of −0.08 eV, and the bond length of H_2_ is 0.814 Å, which is much longer than free molecular H_2_ (0.752 Å) in the gas phase. While H_2_ molecule is adsorbed on Co atom of Co_1_/NC with adsorption energy of 0.033 eV, and the bond length of H_2_ is 0.783 Å. The much longer H-H bond length of adsorbed molecule H_2_ indicates that Co_1_/NPC has a higher activation ability for H_2_ dissociation than Co_1_/NC. Subsequently, one of the H atoms moves to a nearby heteroatom (N and P) to yield heteroatom-H^δ+^, leaving another H atom on Co atom as Co-H^δ-^. Direct dissociation of H_2_ on both Co_1_/NPC and Co_1_/NC in the absence of water, the transition state is almost the same (~1.21 eV), the only difference is that the dissociation of H_2_ on Co_1_/NPC is exothermic by 0.20 eV, while on Co_1_/NPC is endothermic by 1.11 eV, suggesting such dissociation manner on Co_1_-N_4_ site is thermodynamically unfavorable (Supplementary Fig. 22). The above difference in the DFT calculations confirms that Co_1_-N_3_P_1_ exhibits much higher catalytic activity for heterolytic cleavage of H_2_.

Ding et al.^18^. reported that the protic solvents play a dominant role in the case of Co-N-C-catalyzed hydrogenation of nitroarenes, where the solvent-mediated H-shuttling mechanism is crucial in the reaction pathway. Compared to the intrinsic hydrogen transfer, the protic solvent-mediated one usually possesses a lower activation barrier, leading to an enhancement of hydrogenation activity in the presence of water or alcohol^49^. Indeed, both Co_1_/NC and Co_1_/NPC show the best activities under ethanol/water solvent and significantly decreased activities in an aprotic solvent such as toluene, acetonitrile, THF, and n-hexane (Supplementary Fig. 23). Further DFT calculations suggest that the activation energy barriers with water-mediated H-shuttling mechanism for the heterolytic cleavage of H_2_ are lower by about 0.16 and 0.01 eV than that through direct dissociation on Co_1_-N_3_P_1_ and Co_1_-N_4_ sites, respectively (Fig. 4c, d). Both kinetic and thermodynamic results suggest that the dissociative activation of H_2_ with help of H_2_O is more favorable to Co_1_/NPC catalyst.

**Fig. 5:**
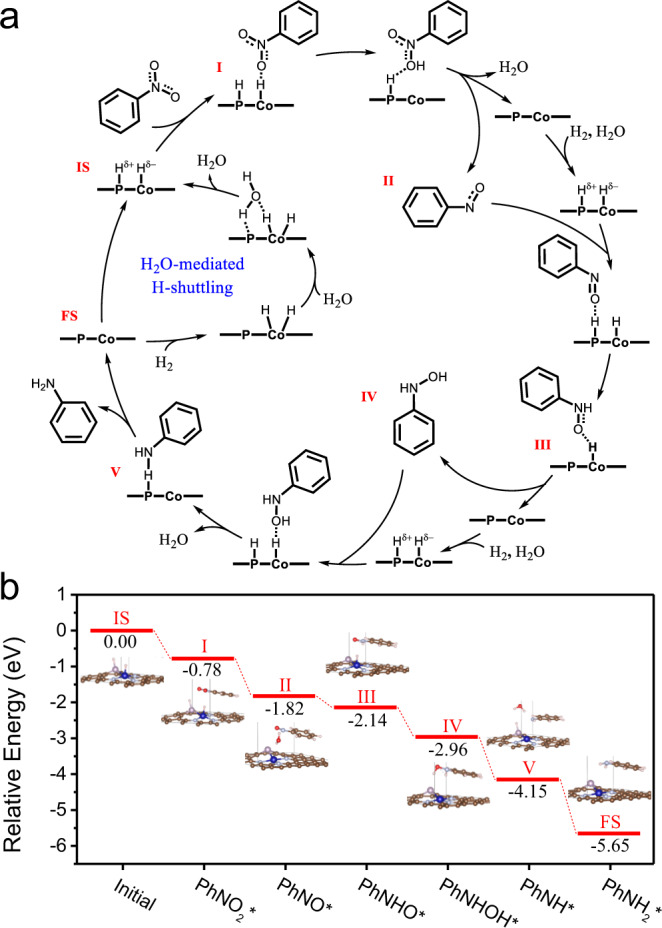
Reaction mechanism of the nitrobenzene reduction. **a** The proposed reaction pathway for the hydrogenation of nitrobenzene to aniline at Co-P interface site. **b** Energy profile of hydrogenation of nitrobenzene over Co_1_-N_3_P_1_ site.

According to the previous reports, the hydrogenation reduction of nitrobenzene to aniline follows the Haber mechanism^7^, namely, PhNO_2_* → PhNOOH* → PhNO* → PhNOH* → PhNHOH* → PhNH* → PhNH_2_*. Based on the above results and reported mechanism in literature, the reaction pathway for hydrogenation of nitrobenzene over Co_1_-N_3_P_1_ catalyst is further proposed by virtue of DFT calculations, as shown in Fig. 5 and Supplementary Fig. 24. One H_2_ molecule first goes through heterolytic cleavage with the assistance of the H_2_O-mediated H-shuttling mechanism to form Co-H^δ-^ and P-H^δ+^ at Co_1_-N_3_P_1_ sites, which can serve as the initial state for the hydrogenation process (Fig. 5a and Supplementary Fig. 25). Then, the target nitrobenzene molecule was adsorbed on the Co_1_-N_3_P_1_ site with a free energy of −0.78 eV (Fig. 5b, I). Subsequently, the activated H atom on Co-H^δ-^ and the O atom of PhNO_2_ are combined to produce PhNOOH intermediate, which is later reduced to PhNO intermediate by the H atom transfer from P-H^δ+^ (II). Notably, the PhNO intermediate can be detected during the reaction process (Supplementary Fig. 26). After that, another H_2_ molecule is dissociated to form an activated H atom, which attacks the oxygen atom of PhNO and reduces it to PhNHOH (III, IV). It is worth noting that the adsorption energies of intermediates PhNO and PhNHOH on the Co_1_-N_3_P_1_ site are more favorable than Ph-NO_2_, ensuring the reaction progress of the targeted substrate (Supplementary Fig. 27). In the next step, the third H_2_ molecule participates in and the formed H atom interacts with PhNHOH to generate the final PhNH_2_ product (V, FS). It can be seen the whole process is highly endothermic, confirming the possibility of the proposed reaction path.

### Substrate exploration and catalytic stability

**Fig. 6:**
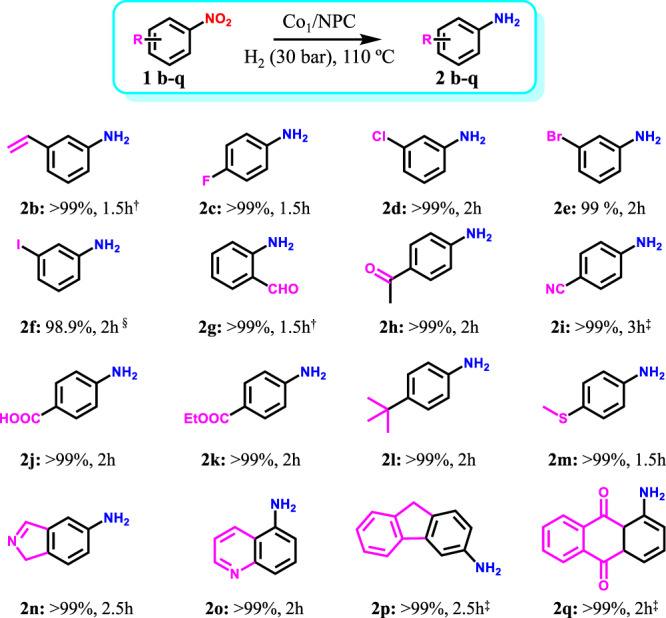
Substrate scope of hydrogenation over the Co_1_/NPC catalyst. Reaction conditions: 1 mmol nitroarenes; 10 mL EtOH; 5 mg catalyst; 110 °C; 3 MPa H_2_. ^†^EtOH/H_2_O, v-v = 4:1, 10 mL; ^§^H_2_, 2 MPa; ^‡^temperature, 120 °C. In all cases, complete conversions of nitroarenes were observed.

A broad scope of substituted nitroarenes was tested to examine the chemoselectivity in nitroarene hydrogenation (Fig. 6). Co_1_/NPC shows impressive chemoselectivity toward the substituted nitroarenes in the presence of other sensitive reducible groups, such as alkenyl (99.7%, 2b), halogen (>98.9%, 2c–f), ketones (>99%, 2g, h), nitrile groups (>99%, 2i), etc. Notably, Co_1_/NPC also exhibits high activity and selectivity toward heterocyclic nitro-compounds (>99%, 2n–q). The superior high selectivity to corresponding anilines is ascribed to the unique character of metal SACs, where there is only one single metal atom for adsorption and activation of substrates.

Furthermore, Co_1_/NPC exhibited tolerable stability. As shown in Supplementary Fig. 28, a slight decrease of activity is observed after five cycles with Co_1_/NPC, which can be ascribed to the loss of some catalysts and active Co species during the recycling experiments (Supplementary Table 1). The AC HAADF-STEM image and Co K-edge EXAFS spectrum of spent Co_1_/NPC indicate that the atomically dispersed Co species is well preserved after five cycles (Supplementary Fig. 29). All these results demonstrate that the Co_1_/NPC catalyst with unsymmetrical Co_1_-N_3_P_1_ configuration possesses unprecedented high activity, high selectivity, and good stability to a wide scope of substrates for hydrogenation of nitroarenes.

## Discussion

In summary, we produced an atomically dispersed Co_1_/NPC catalyst with an unsymmetrically Co_1_-N_3_P_1_ coordination structure. Due to the increased electron density and upshift d-band center of Co atoms in Co_1_-N_3_P_1_, H_2_ dissociation was proved to be more favorable, resulting in much enhanced catalytic activity. In nitrobenzene hydrogenation reaction, the as-prepared Co_1_-N_3_P_1_ SAC exhibited a 60-fold higher TOF value (6560 h^−1^) than that of Co_1_-N_4_ SAC and more than tenfold higher than the state-of-the-art M_1_-N_x_-C SACs in literature. In addition, Co_1_-N_3_P_1_ SAC also displayed superior selectivity (>99%) towards the substituted nitroarenes with the co-existence of other sensitive reducible groups. This work provides new insight into rationally modulating the coordination structure of central metal atoms for boosting the catalytic performance of SACs in heterogeneous catalysis.

## Methods

### Synthesis of Co_1_/NPC and Co_1_/NC

In a typical procedure, Co(NO_3_)_2_·6H_2_O (6.5 mg), tannic acid (TA, 500 mg), and (2-Aminoethyl) phosphonic acid (AePA, 126 mg) were dissolved into 30 mL DI water at 100 °C (marked as solution A). g-C_3_N_4_ nanosheets (1 g) were dispersed well in 100 mL DI water with ultrasound (marked as solution B). Then, solution A was added dropwise into solution B with a strong stirring at 100 °C until the mixed system was forced to yield a slurry. Subsequently, the obtained powder after freeze-dried was pyrolyzed at 900 °C for 2 h under Ar atmosphere. Finally, the as-prepared material was directly used without further treatment, denoted as Co_1_/NPC.

The synthesis process for Co_1_/NC is the same as that of Co_1_/NPC except without the addition AePA.

### Characterizations

The powder X-ray diffraction (XRD) patterns were recorded on a Rigaku D/max-2500n diffractometer with Cu Kα radiation (λ = 1.5418 Å) at 40 kV and 200 mA. The morphologies and microstructures of the samples were measured on the transmission electron microscopy (TEM) (JEM-2100F, JEOL, Japan) and the scanning electron microscopy (SEM) (HITACHI S-4800, Japan). Element mapping was characterized on TEM equipped with Oxford detection. X-ray photoelectron spectroscopy (XPS) measurements were performed on a VG Scientific ESCALab220i-XL electron spectrometer using 300 W Al kα radiation. Inductively coupled plasma atomic emission spectroscopy (ICP-AES) was conducted on a Shimadzu ICPE-9000 to confirm the loading content of metal on the catalysts. The AC HAADF-STEM images were carried out in a JEOL ARM300F at 300 kV, equipped with a probe spherical aberration corrector. Brunauer–Emmett–Teller (BET) surface areas were measured by N_2_ adsorption-desorption isotherms at 77 K with a Micromeritics ASAP 2460 instrument. The HAADF imaging and EELS mapping were both performed using a Nikon HERMES-100 aberration-corrected scanning transmission electron microscope under 60 kV accelerating voltage with a ~22 pA probe. The probe convergence semi-angle, HAADF collection semi-angle, and EELS collection semi-angle is 32 mrad, 75–210 mrad, and 0–75 mrad, respectively.

### XAS measurements and analysis

The cobalt K-edges XAFS spectra of the standards and samples were collected at the beamline 1W1B of the Beijing Synchrotron Radiation Facility (BSRF). The typical energy of the storage ring was 2.5 GeV and the electron current was ~250 mA in the top-up mode. The white light was monochromatized by a Si (111) double-crystal monochromator and calibrated with a Co foil (K-edge at 7709 eV). Samples were pressed into thin slices and positioned at 45° to the incident beam in the sample holder. The XAFS spectra were recorded in fluorescence mode with a Lytle detector oriented at 90° to the incoming beam.

The XAFS data were analyzed using the software packages Demeter^50^. The spectra were normalized using Athena firstly, and then shell fittings were performed with Artemis. The *χ*(k) function was Fourier-transformed (FT) using *k*^3^ weighting, and all fittings were done in R-space. The coordination parameters of samples were obtained by fitting the experimental peaks with theoretical amplitude. The quantitative curve-fittings were conducted with a Fourier transform k-space range of 2.7–11.8 Å^−1^. The backscattering amplitude *F*(k) and phase shift Φ(k) were calculated by the FEFF7.0 code. While the curve-fitting, all the amplitude reduction factor S_0_^2^ was set to the best-fit value of 0.87 determined from fitting the data of cobalt foil by fixing coordination numbers as the known crystallographic value. In order to fit the curves in the R-range of 1.0–2.0 Å, we considered Co-N and Co-P paths as the central-peripheral. For each path, the structural parameters, like coordination number (CN), interatomic distance (R), Debye–Waller factors (σ^2^), and inner potential correction (ΔE_0_) were opened to be varied.

### Catalytic performance evaluation

For the liquid phase hydrogenation of nitrobenzene, substrates (2 mmol), catalyst (5 mg), and solvent (EtOH/H_2_O, v-v = 4:1, 40 mL) were added into a 100 mL high-pressure autoclave. Then the autoclave was flushed three times with H_2_ and charged to certain pressure (3 MPa H_2_). The reaction was performed at the desired temperature. The product was collected at the reserved time and immediately analyzed using gas chromatography in combination with mass spectrometry (Shimadzu GCMS-QP2010S). The TOF values were determined within the substrate conversion below 20%, and the calculation of TOF was based on the total Co amount in catalysts. To evaluate the reusability of catalyst, the samples from the last reaction were separated by centrifugation, washing with ethyl acetate to remove the substrate, and drying under the vacuum. For the kinetic isotope effect test, D_2_ and D_2_O are used instead of H_2_ and H_2_O.

### DFT calculations

All the spin-polarized first-principles calculations have used the code VASP^51–53^. Valence electrons of O(2*s*, 2*p*), N(2*s*, 2*p*), H(1*s*), C(2*s*, 2*p*), P(3*s*, 3*p*), and Co(3*d*, 4 *s*) were treated on a basis of plane waves explicitly^54^, while the core electrons were described with the projector-augmented wave method^55^. Spin-polarized calculations were carried out at the level of the generalized gradient approximation (GGA) adopting the Perdew, Burke, and Ernzerhof (PBE) exchange-correlation functional^56^. A kinetic energy cutoff of 400 eV was used for all calculations. The truncation criteria for the electronic and ionic loops were 10^−5^ eV and 10^−2^ eV/Å, respectively. Long-range dispersion was included according to the D_3_ method introduced by Grimme^54^. The vacuum layer was set to 20 Å to avoid interaction from adjacent cells. All the transition states were determined by using the climbing image nudged elastic band (CINEB) method^57,58^ and transition states were characterized via frequency analysis to ensure a single imaginary frequency in the desired reaction direction. The pure graphene is modeled by a (7 × 7) supercell with 49 carbon atoms, and the Co-N_x_P_y_-Gr model is modeled by one cobalt atom adsorption at vacancy site which is composed by getting rid of two carbon atoms and the N and P atoms substitute for four carbon atoms around cobalt atom, respectively. Monkhorst–Pack (5 × 5 × 1) Γ-centered grid sampling for the Brillouin zone was used for geometry optimization, and dipole corrections were included in the z-direction for each model surface. To study the stability of Co binding on graphene with N, P dual-coordination, the formation energy, and adsorption energy were defined as follows:

1 $${{{{{{\rm{E}}}}}}}_{{{{{{\rm{for}}}}}}}={{{{{\rm{E}}}}}}({{{{{\rm{Co}}}}}}\mbox{-}{{{{{{\rm{N}}}}}}}_{{{{{{\rm{x}}}}}}}{{{{{{\rm{P}}}}}}}_{{{{{{\rm{y}}}}}}}\mbox{-}{{{{{\rm{Gr}}}}}})-{{{{{\rm{E}}}}}}({{{{{{\rm{N}}}}}}}_{{{{{{\rm{x}}}}}}}{{{{{{\rm{P}}}}}}}_{{{{{{\rm{y}}}}}}})-{{{{{\rm{E}}}}}}({{{{{\rm{Co}}}}}}\mbox{-}{{{{{\rm{bulk}}}}}})$$

2 $${{{{{{\rm{E}}}}}}}_{{{{{{\rm{ads}}}}}}}={{{{{\rm{E}}}}}}({{{{{\rm{Co}}}}}}\mbox{-}{{{{{{\rm{N}}}}}}}_{{{{{{\rm{x}}}}}}}{{{{{{\rm{P}}}}}}}_{{{{{{\rm{y}}}}}}}\mbox{-}{{{{{\rm{Gr}}}}}})-{{{{{\rm{E}}}}}}({{{{{{\rm{N}}}}}}}_{{{{{{\rm{x}}}}}}}{{{{{{\rm{P}}}}}}}_{{{{{{\rm{y}}}}}}})-{{{{{\rm{E}}}}}}({{{{{\rm{Co}}}}}})$$

In the equations, E(Co-N_x_P_y_-Gr) is the total energy of adsorbed systems, E(N_x_P_y_) is the energy of graphene with doped N and P, E(Co-bulk) is the energy of one atom in the most stable Co crystal, and E(Co) is the energy of cobalt in the gas phase.

## Supplementary information

Supplementary Information

Peer Review File

## Data Availability

The data supporting the findings of this study are available within the article and its Supplementary Information. Source data are provided with this paper. Additional data are available from the corresponding authors on reasonable request.

## References

[1] Jagadeesh RV (2013). Nanoscale Fe_2_O_3_-based catalysts for selective hydrogenation of nitroarenes to anilines. Science.

[2] Zhang LL, Zhou MX, Wang AQ, Zhang T (2020). Selective hydrogenation over supported metal catalysts: from nanoparticles to single atoms. Chem. Rev..

[3] Lang R (2020). Single-atom catalysts based on the metal-oxide interaction. Chem. Rev..

[4] Yan H (2018). Atomic engineering of high-density isolated Co atoms on graphene with proximal-atom controlled reaction selectivity. Nat. Commun..

[5] Wei H (2014). FeO_x_-supported platinum single-atom and pseudo-single-atom catalysts for chemoselective hydrogenation of functionalized nitroarenes. Nat. Commun..

[6] Ye T-N (2020). Stable single platinum atoms trapped in sub-nanometer cavities in 12CaO•7Al_2_O_3_ for chemoselective hydrogenation of nitroarenes. Nat. Commun..

[7] Zhang S (2016). High catalytic activity and chemoselectivity of sub-nanometric Pd clusters on porous nanorods of CeO_2_ for hydrogenation of nitroarenes. J. Am. Chem. Soc..

[8] Boronat M (2007). A molecular mechanism for the chemoselective hydrogenation of substituted nitroaromatics with nanoparticles of gold on TiO_2_ catalysts: a cooperative effect between gold and the support. J. Am. Chem. Soc..

[9] Serna P, Corma A (2015). Transforming nano metal nonselective particulates into chemoselective catalysts for hydrogenation of substituted nitrobenzenes. ACS Catal..

[10] Yang X-F (2013). Single-atom catalysts: a new frontier in heterogeneous catalysis. Acc. Chem. Res..

[11] Fu T (2014). Acid-resistant catalysis without use of noble metals: carbon nitride with underlying nickel. ACS Catal..

[12] Westerhaus FA (2013). Heterogenized cobalt oxide catalysts for nitroarene reduction by pyrolysis of molecularly defined complexes. Nat. Chem..

[13] Wang Y (2020). Chemoselective hydrogenation of nitroaromatics at the nanoscale iron(III)-OH-platinum interface. Angew. Chem. Int. Ed..

[14] Li H (2019). Cobalt single atoms anchored on N-doped ultrathin carbon nanosheets for selective transfer hydrogenation of nitroarenes. Sci. China Mater..

[15] Zhang L (2020). Atomically dispersed Co catalyst for efficient hydrodeoxygenation of lignin-derived species and hydrogenation of nitroaromatics. ACS Catal..

[16] Zhou P (2017). High performance of a cobalt-nitrogen complex for the reduction and reductive coupling of nitro compounds into amines and their derivatives. Sci. Adv..

[17] Liu W (2016). Single-atom dispersed Co-N-C catalyst: structure identification and performance for hydrogenative coupling of nitroarenes. Chem. Sci..

[18] Li M (2021). Origin of the activity of Co-N-C catalysts for chemoselective hydrogenation of nitroarenes. ACS Catal..

[19] Zhou D (2021). Tuning the coordination environment of single-atom catalyst M-N-C towards selective hydrogenation of functionalized nitroarenes. Nano Res..

[20] Wan J (2020). In situ phosphatizing of triphenylphosphine encapsulated within metal-organic frameworks to design atomic Co_1_-P_1_N_3_ interfacial structure for promoting catalytic performance. J. Am. Chem. Soc..

[21] Wei X (2020). Cross-linked polyphosphazene hollow nanosphere-derived N/P-doped porous carbon with single nonprecious metal atoms for the oxygen reduction reaction. Angew. Chem. Int. Ed..

[22] Long X (2020). Graphitic phosphorus coordinated single Fe atoms for hydrogenative transformations. Nat. Commun..

[23] Yuan K (2020). Boosting oxygen reduction of single iron active sites via geometric and electronic engineering: nitrogen and phosphorus dual coordination. J. Am. Chem. Soc..

[24] Ren Y (2019). Unraveling the coordination structure-performance relationship in Pt_1_/Fe_2_O_3_ single-atom catalyst. Nat. Commun..

[25] Liu J (2021). Direct observation of metal oxide nanoparticles being transformed into metal single atoms with oxygen-coordinated structure and high-loadings. Angew. Chem. Int. Ed..

[26] Wang L (2019). A sulfur-tethering synthesis strategy toward high-loading atomically dispersed noble metal catalysts. Sci. Adv..

[27] Zhang J (2019). Tuning the coordination environment in single-atom catalysts to achieve highly efficient oxygen reduction reactions. J. Am. Chem. Soc..

[28] Shang H (2020). Engineering unsymmetrically coordinated Cu-S_1_N_3_ single atom sites with enhanced oxygen reduction activity. Nat. Commun..

[29] Hai X (2020). Engineering local and global structures of single Co atoms for a superior oxygen reduction reaction. ACS Catal..

[30] Chen C (2021). Zero-valent palladium single-atoms catalysts confined in black phosphorus for efficient semi-hydrogenation. Adv. Mater..

[31] Chen Y (2021). Atomic-level modulation of electronic density at cobalt single-atom sites derived from metal-organic frameworks: enhanced oxygen reduction performance. Angew. Chem. Int. Ed..

[32] Ji S (2021). Matching the kinetics of natural enzymes with a single-atom iron nanozyme. Nat. Catal..

[33] Zhang Y, Jiao L, Yang W, Xie C, Jiang H-L (2021). Rational fabrication of low-coordinate single-atom Ni electrocatalysts by MOFs for highly selective CO_2_ reduction. Angew. Chem. Int. Ed..

[34] Zhang F (2017). In situ mosaic strategy generated Co-based N-doped mesoporous carbon for highly selective hydrogenation of nitroaromatics. J. Catal..

[35] Qu Y, Yang H, Wang S, Chen T, Wang G (2017). Hydrogenation of nitrobenzene to aniline catalyzed by C-60-stabilized Ni. Catal. Commun..

[36] He, J. et al. Strategic defect engineering of metal-organic frameworks for optimizing the fabrication of single-atom catalysts. *Adv. Funct. Mater*. **31**, 2103597 (2021).

[37] Wang H (2020). Highly efficient hydrogenation of nitroarenes by N-doped carbon-supported cobalt single-atom catalyst in ethanol/water mixed solvent. ACS Appl. Mater. Interfaces.

[38] Cai Q (2021). Boosted catalytic hydrogenation performance using isolated Co sites anchored on nitrogen-incorporated hollow porous carbon. J. Phys. Chem. C..

[39] Sun X (2018). Single cobalt sites in mesoporous N-doped carbon matrix for selective catalytic hydrogenation of nitroarenes. J. Catal..

[40] Sun X (2017). Metal-organic framework mediated cobalt/nitrogen-doped carbon hybrids as efficient and chemoselective catalysts for the hydrogenation of nitroarenes. ChemCatChem.

[41] Tian S (2021). Single-atom Fe with Fe_1_N_3_ structure showing superior performances for both hydrogenation and transfer hydrogenation of nitrobenzene. Sci. China Mater..

[42] Yang F (2019). Atomically dispersed Ni as the active site towards selective hydrogenation of nitroarenes. Green. Chem..

[43] Wang X, Li Y (2016). Chemoselective hydrogenation of functionalized nitroarenes using MOF-derived Co-based catalysts. J. Mol. Catal. A: Chem..

[44] Kuai L (2020). Titania supported synergistic palladium single atoms and nanoparticles for room temperature ketone and aldehydes hydrogenation. Nat. Commun..

[45] Liu P (2016). Photochemical route for synthesizing atomically dispersed palladium catalysts. Science.

[46] Bai L (2016). Explaining the size dependence in platinum-nanoparticle-catalyzed hydrogenation reactions. Angew. Chem. Int. Ed..

[47] Qin R (2020). Alkali ions secure hydrides for catalytic hydrogenation. Nat. Catal..

[48] Liu W (2018). A durable nickel single-atom catalyst for hydrogenation reactions and cellulose valorization under harsh conditions. Angew. Chem. Int. Ed..

[49] Merte LR (2012). Water-mediated proton hopping on an iron oxide surface. Science.

[50] Ravel B, Newville M (2005). ATHENA, ARTEMIS, HEPHAESTUS: data analysis for X-ray absorption spectroscopy using IFEFFIT. J. Synchrotron Radiat..

[51] Kresse G, Joubert D (1999). From ultrasoft pseudopotentials to the projector augmented-wave method. Phys. Rev. B.

[52] Kresse G, Furthmuller J (1996). Efficiency of ab-initio total energy calculations for metals and semiconductors using a plane-wave basis set. Comp. Mater. Sci..

[53] Kresse G, Furthmuller J (1996). Efficient iterative schemes for ab initio total-energy calculations using a plane-wave basis set. Phys. Rev. B.

[54] Grimme S, Antony J, Ehrlich S, Krieg H (2010). A consistent and accurate ab initio parametrization of density functional dispersion correction (DFT-D) for the 94 elements H-Pu. J. Chem. Phys..

[55] Blochl PE (1994). Projector augmented-wave method. Phys. Rev. B.

[56] Perdew JP, Burke K, Ernzerhof M (1996). Generalized gradient approximation made simple. Phys. Rev. Lett..

[57] Henkelman G, Uberuaga BP, Jonsson H (2000). A climbing image nudged elastic band method for finding saddle points and minimum energy paths. J. Chem. Phys..

[58] Henkelman G, Jonsson H (2000). Improved tangent estimate in the nudged elastic band method for finding minimum energy paths and saddle points. J. Chem. Phys..

