# Supplementary material for: Unprecedentedly high activity and selectivity for hydrogenation of nitroarenes with single atomic Co1-N3P1 sites
Source: Nat Commun. 2022 Feb 7;13:723. doi: 10.1038/s41467-022-28367-9 (PMC8821636; doi:10.1038/s41467-022-28367-9)
Supplement: Supplementary file 1 — Supplementary Information [file 41467_2022_28367_MOESM1_ESM.pdf]

Supplementary Information

Unprecedentedly high activity and selectivity for hydrogenation of nitroarenes with single atomic Co_1−N_3P_1 sites

Hongqiang Jin,[a, b] Peipei Li,[a, b] Peixin Cui,[c] Jinan Shi,[d] Wu Zhou,[d] Xiaohu Yu,*[e] Weiguo Song[a, b] and Changyan Cao*[a, b]

[a]Beijing National Laboratory for Molecular Sciences, CAS Research/Education Center for Excellence in Molecular Sciences, Laboratory of Molecular Nanostructures and Nanotechnology, Institute of Chemistry, Chinese Academy of Sciences, Beijing 100190, China

E-mail: cycao@iccas.ac.cn

[b]School of Chemical Sciences, University of Chinese Academy of Sciences, Beijing 100049, China

[c]Key Laboratory of Soil Environment and Pollution Remediation, Institute of Soil Science, Chinese Academy of Sciences, Nanjing 210008, China

[d]School of Physical Sciences and CAS Key Laboratory of Vacuum Physics, University of Chinese Academy of Sciences, Beijing 100049, China; CAS Center for Excellence in Topological Quantum Computation, University of Chinese Academy of Sciences, Beijing 100049, China

[e]Institute of Theoretical and Computational Chemistry, Shaanxi Key Laboratory of Catalysis, School of Chemical & Environment Sciences, Shaanxi University of Technology, Hanzhong 723000, China

E-mail: yuxiaohu@snut.edu.cn

Supplementary Figures and Tables

Supplementary Fig. 1 Schematic illustration of the preparation of Co_1/NPC.

Supplementary Fig. 2 FTIR spectra of Co_1/NPC.

Supplementary Fig. 3 Raman spectra of Co_1/NC and Co_1/NPC.

Supplementary Fig. 4 XRD patterns of Co_1/NC and Co_1/NPC.

Supplementary Fig. 5 TEM, HAADF-STEM and EDS mapping of Co_2P NPs/C.

Supplementary Fig. 6 SEM images of Co_1/NPC and Co_1/NC.

Supplementary Fig. 7 TEM and HRTEM images of Co_1/NPC and Co_1/NC.

Supplementary Fig. 8 HAADF-STEM, EDS mapping and EDX spectra of Co_1/NPC.

Supplementary Fig. 9 EDS mapping of Co_1/NC.

Supplementary Fig. 10 AC HAADF-STEM image of Co_1/NC.

Supplementary Fig. 11 N_2 adsorption/desorption isotherms and pore size distribution.

Supplementary Fig. 12 N *1s* XPS spectrum of Co_1/NC.

Supplementary Fig. 13 P *2p* XPS spectra of Co_1/NPC-800 sample.

Supplementary Fig. 14 k^3-weight FT-EXAFS fitting curves of Co_1/NPC and Co_1/NC.

Supplementary Fig. 15 EXAFS fitting in k space.

Supplementary Fig. 16 The calculated XANES data of Co_1-N_4 site.

Supplementary Fig. 17 The side/top views and the formation energies of Co_1-N_1P_3, Co_1-N_2P_2 and Co_1-N_3P_1 structures.

Supplementary Fig. 18 Comparison of samples for nitrobenzene conversion.

Supplementary Fig. 19 Time course of nitrobenzene conversions over Co_1/NPC sample.

Supplementary Fig. 20 Hydrogenation of nitrobenzene under different temperatures.

Supplementary Fig. 21 The initial states and final states for the direct dissociation of H_2 in the absence of water.

Supplementary Fig. 22 Energies profiles for the direct H_2 dissociation pathways in the absence of water.

Supplementary Fig. 23 Catalytic performance in different solvents.

Supplementary Fig. 24 The optimization process of the reaction path of nitrobenzene hydrogenation over Co_1/NPC sample.

Supplementary Fig. 25 The pathways for the H_2O-mediated H-shuttling dissociation of hydrogen.

Supplementary Fig. 26 GC-MS results of the mixture compositions during the reaction.

Supplementary Fig. 27 The adsorption energies of Ph-NO_2, Ph-NHOH and Ph-NO on Co_1-N_3P_1.

Supplementary Fig. 28 Stability test of Co_1/NPC.

Supplementary Fig. 29 EDS mapping, HAADF-STEM image and EXAFS spectra of Co_1/NPC-spent sample.

Supplementary Table 1. Co contents estimated from ICP.

Supplementary Table 2. EXAFS fitting parameters at the Co K-edge for various samples.

Supplementary Table 3. Comparison of hydrogenation of nitroarenes.

Supplementary Table 4. Kinetic parameters under different reaction temperatures.

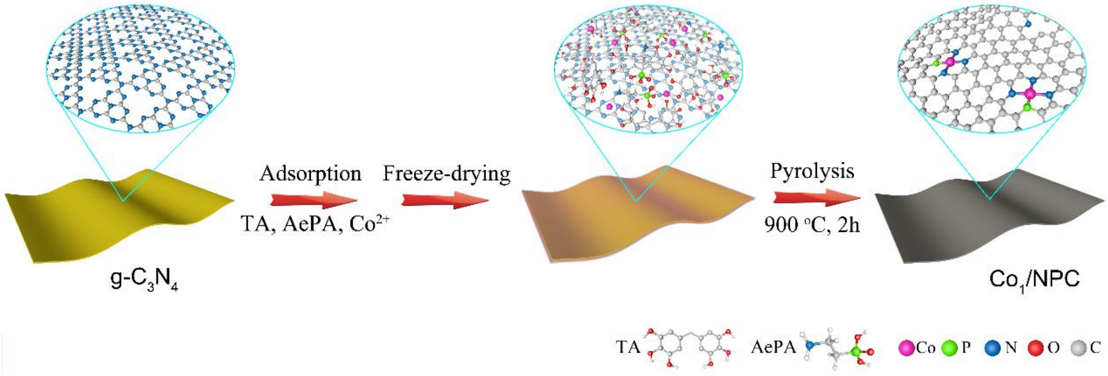

Supplementary Fig. 1 Schematic illustration of the preparation process for Co_1/NPC catalyst.

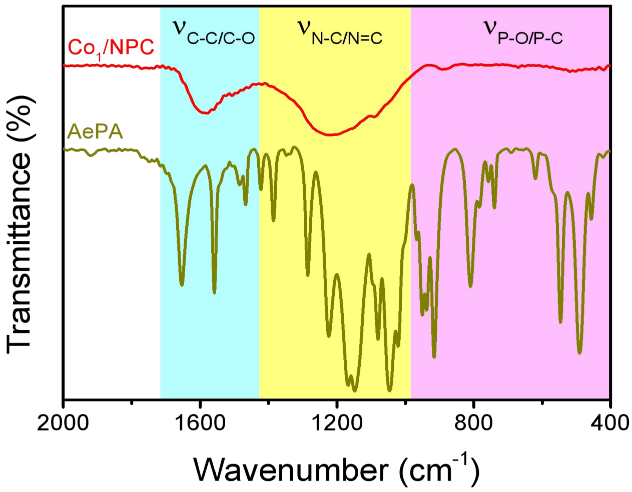

Supplementary Fig. 2 FTIR spectra of AePA and Co_1/NPC sample.

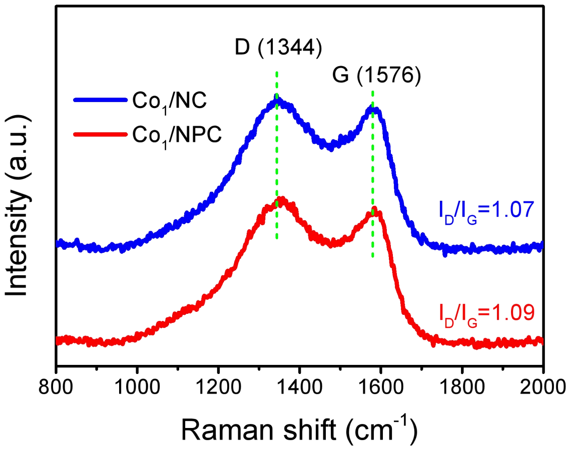

Supplementary Fig. 3 Raman spectra of Co_1/NC and Co_1/NPC.

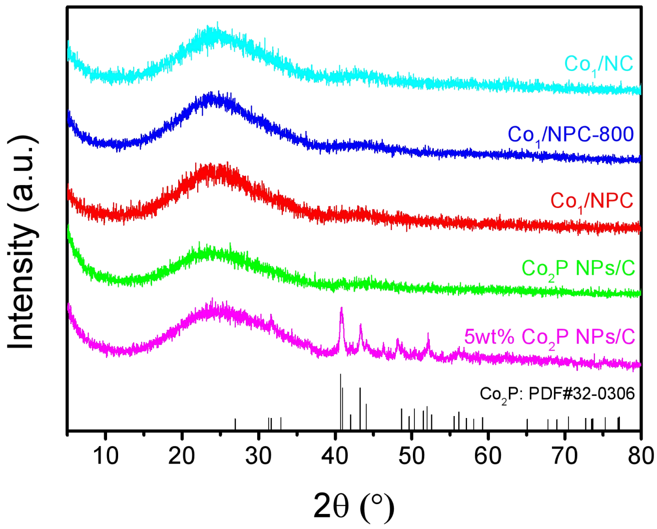

Supplementary Fig. 4 XRD patterns of Co_1/NC, Co_1/NPC, Co_2P NPs/C and Co_2P standard card.

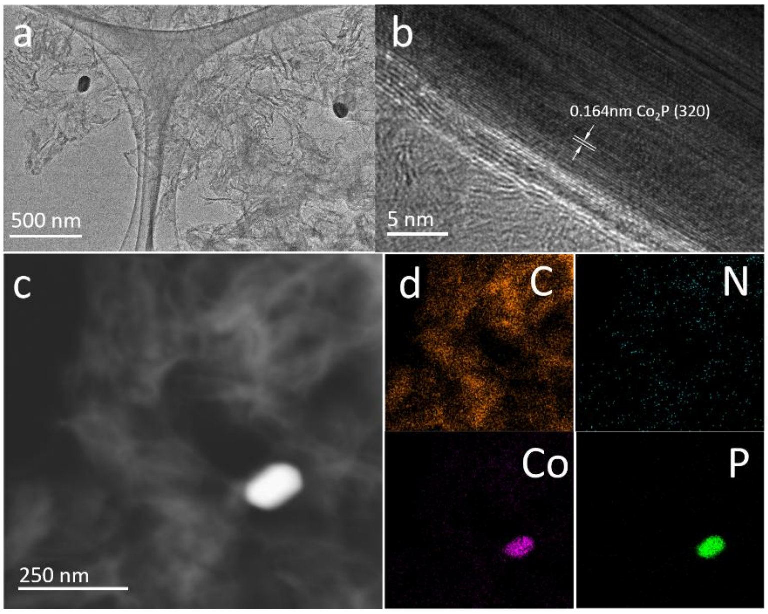

Supplementary Fig. 5 (a) TEM and (b) HRTEM images of Co_2P NPs/C. (c) HAADF-STEM image and (d) corresponding EDS mapping distribution of Co_2P NPs/C.

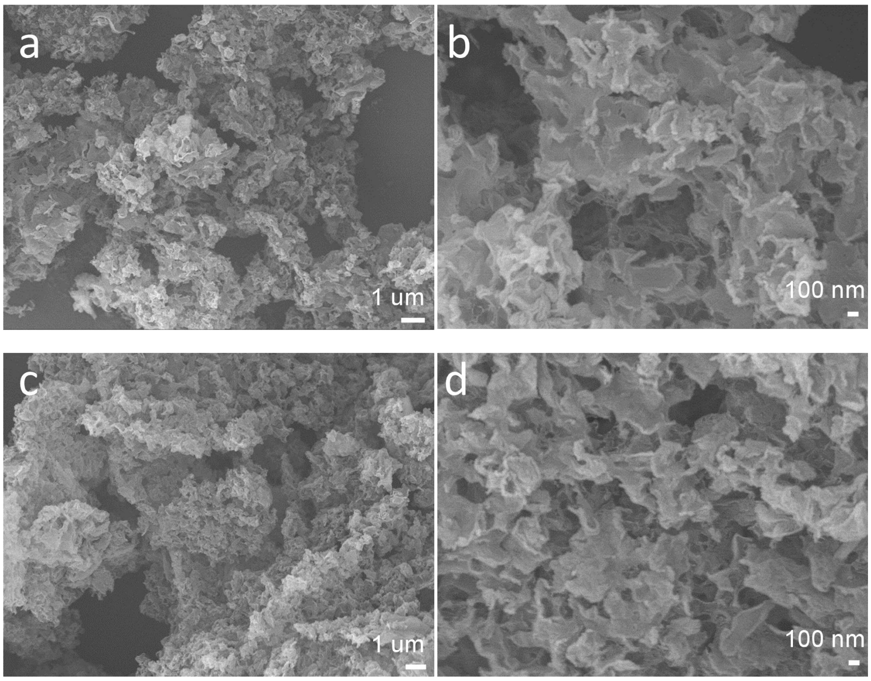

Supplementary Fig. 6 SEM images of (a-b) Co_1/NPC and (c-d) Co_1/NC.

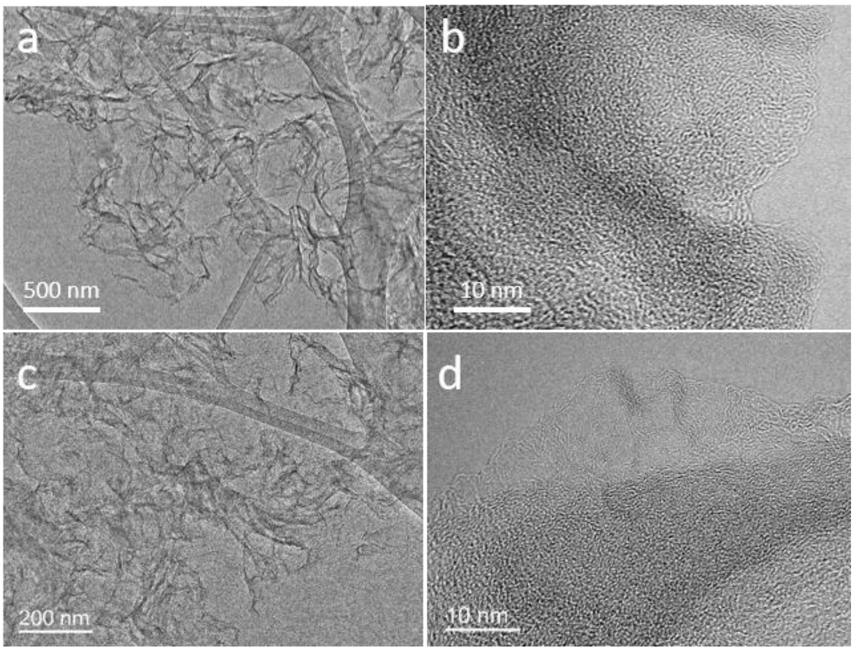

Supplementary Fig. 7 (a) TEM and (b) HRTEM images of Co_1/NPC. (c) TEM and (d) HRTEM images of Co_1/NC.

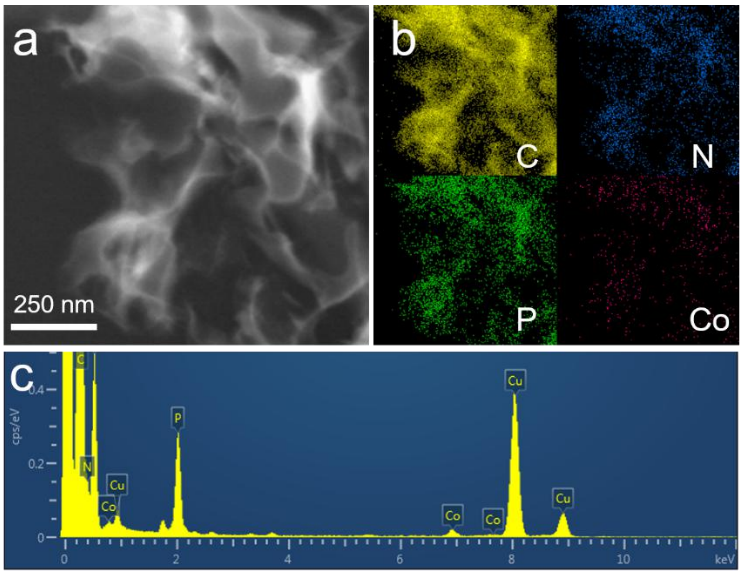

Supplementary Fig. 8 (a) HAADF-STEM image and (b) corresponding EDS mapping distribution of Co_1/NPC for C (yellow), N (blue), P (light green) and Co (purple). (c) EDX spectra of Co_1/NPC.

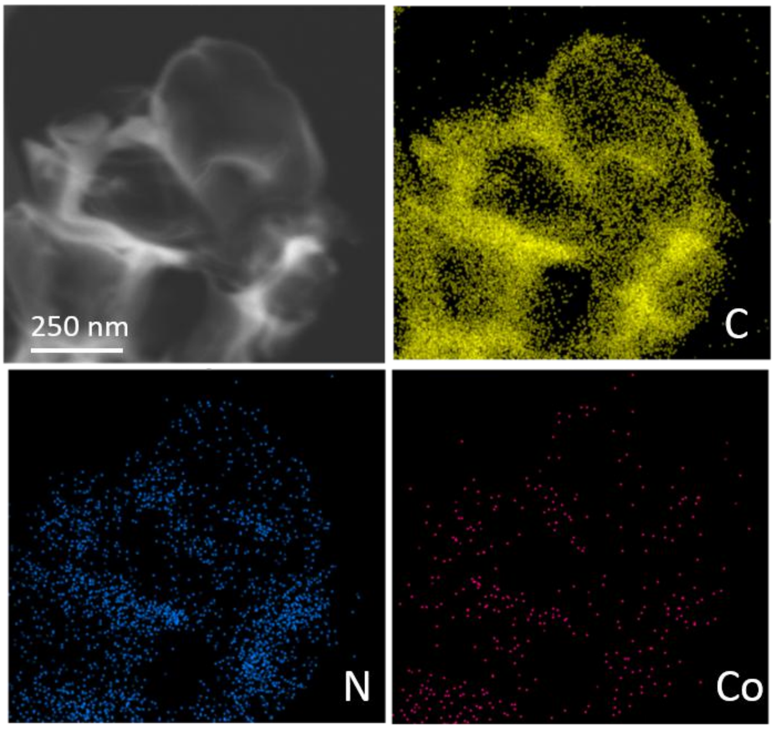

Supplementary Fig. 9 HAADF-STEM image and corresponding EDS mapping distribution of Co_1/NC for C (yellow), N (blue), and Co (purple).

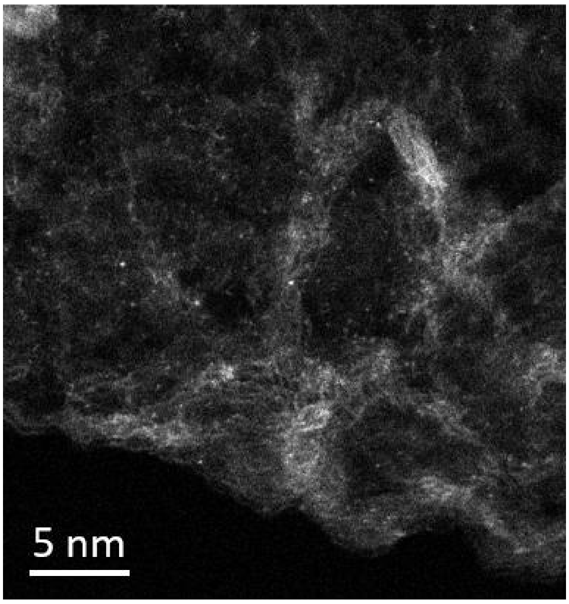

Supplementary Fig. 10 AC HAADF-STEM image of Co_1/NC.

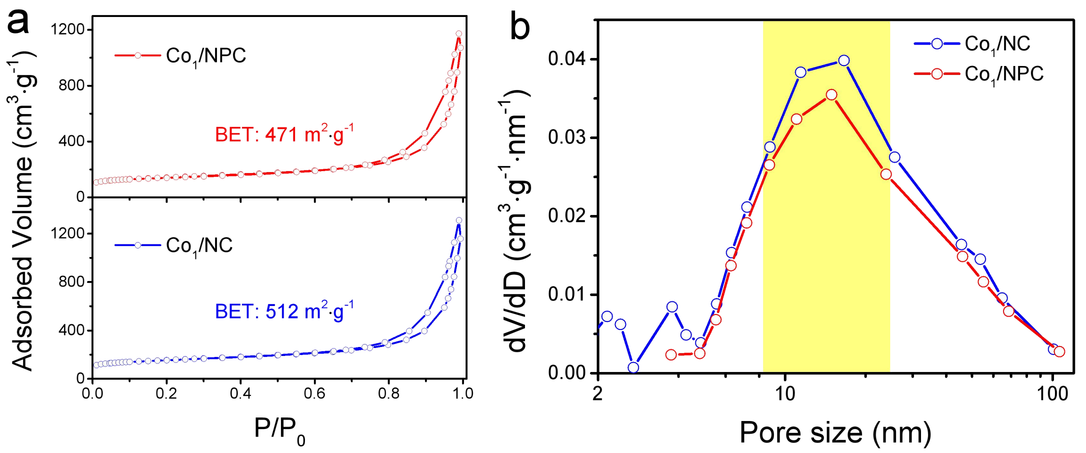

Supplementary Fig. 11 (a) N_2 adsorption/desorption isotherms and (b) pore size distributions for Co_1/NPC (red) and Co_1/NC (blue).

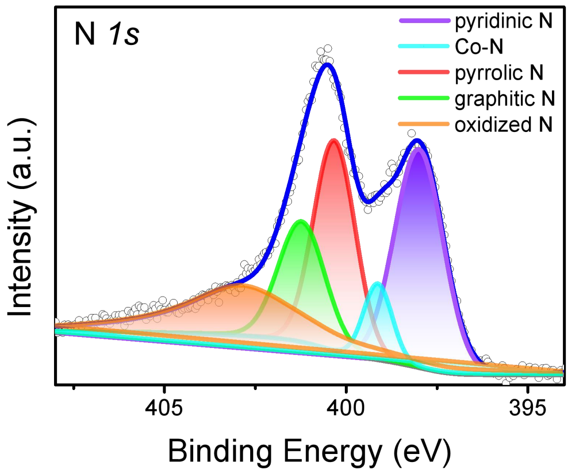

Supplementary Fig. 12 N *1s* XPS spectrum of Co_1/NC.

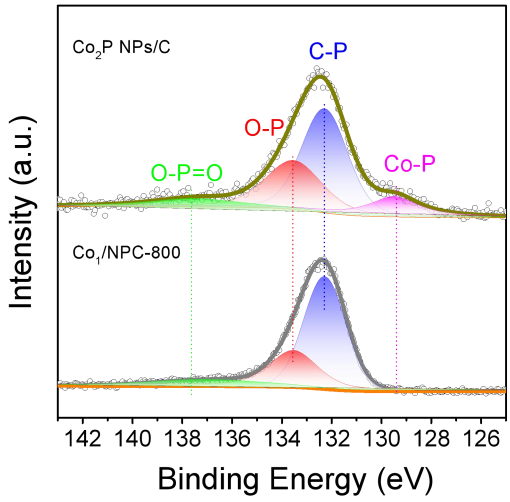

Supplementary Fig. 13 P *2p* XPS spectra of Co_1/NPC-800 and Co_2P NPs/C samples.

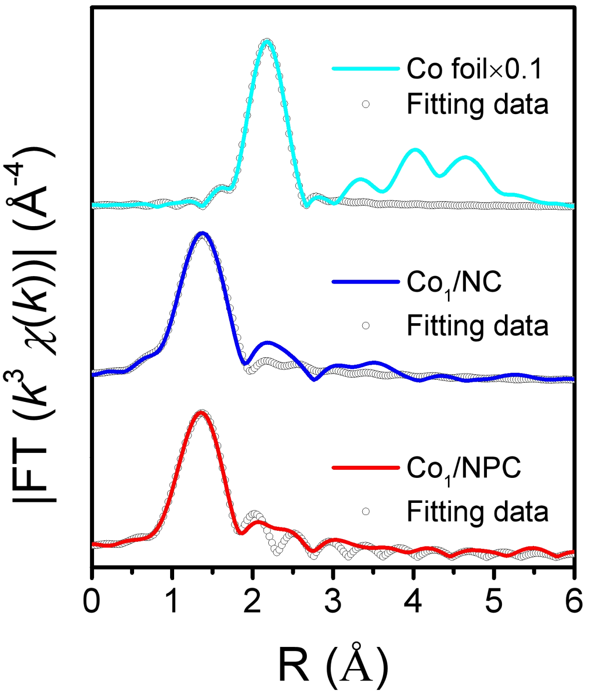

Supplementary Fig. 14 k^3-weight FT-EXAFS fitting curves of Co_1/NPC, Co_1/NC, and Co foil.

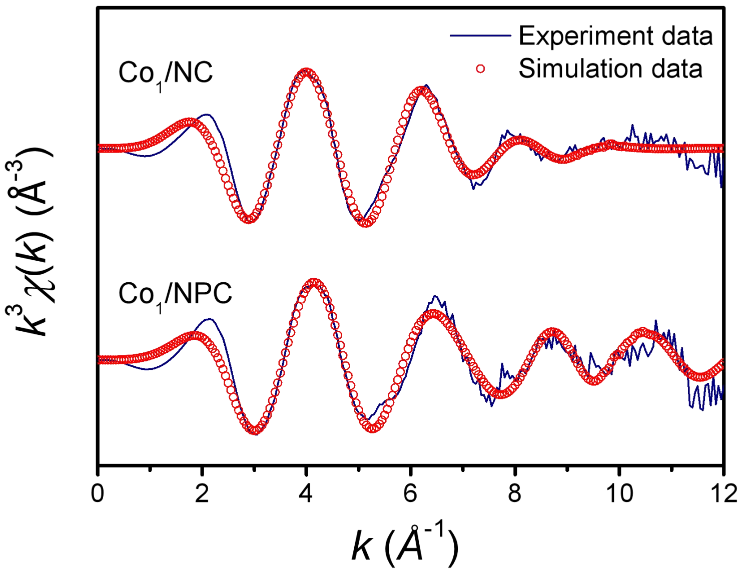

Supplementary Fig. 15 EXAFS fitting analysis of Co_1/NC and Co_1/NPC in k space.

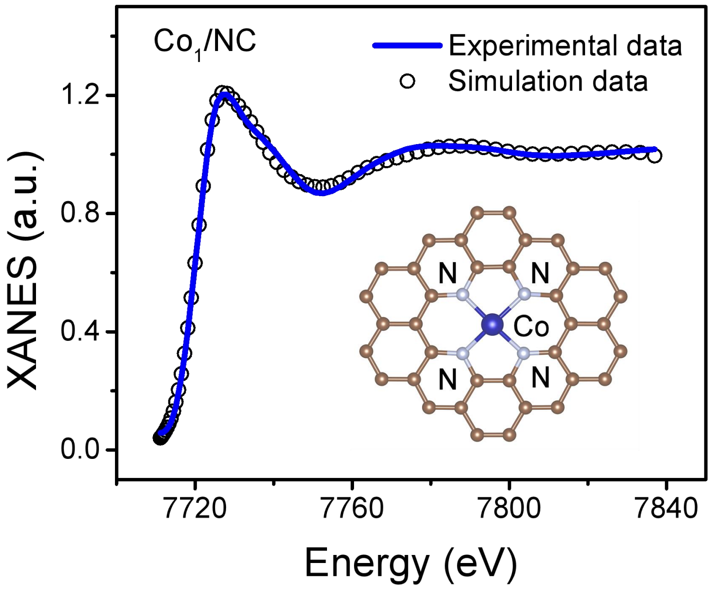

Supplementary Fig. 16 The experimental XANES curve in comparison with the calculated XANES data of Co_1-N_4 site in Co_1/NC sample. Inset: the schematic atomic structure of Co_1/NC derived from the EXAFS results.

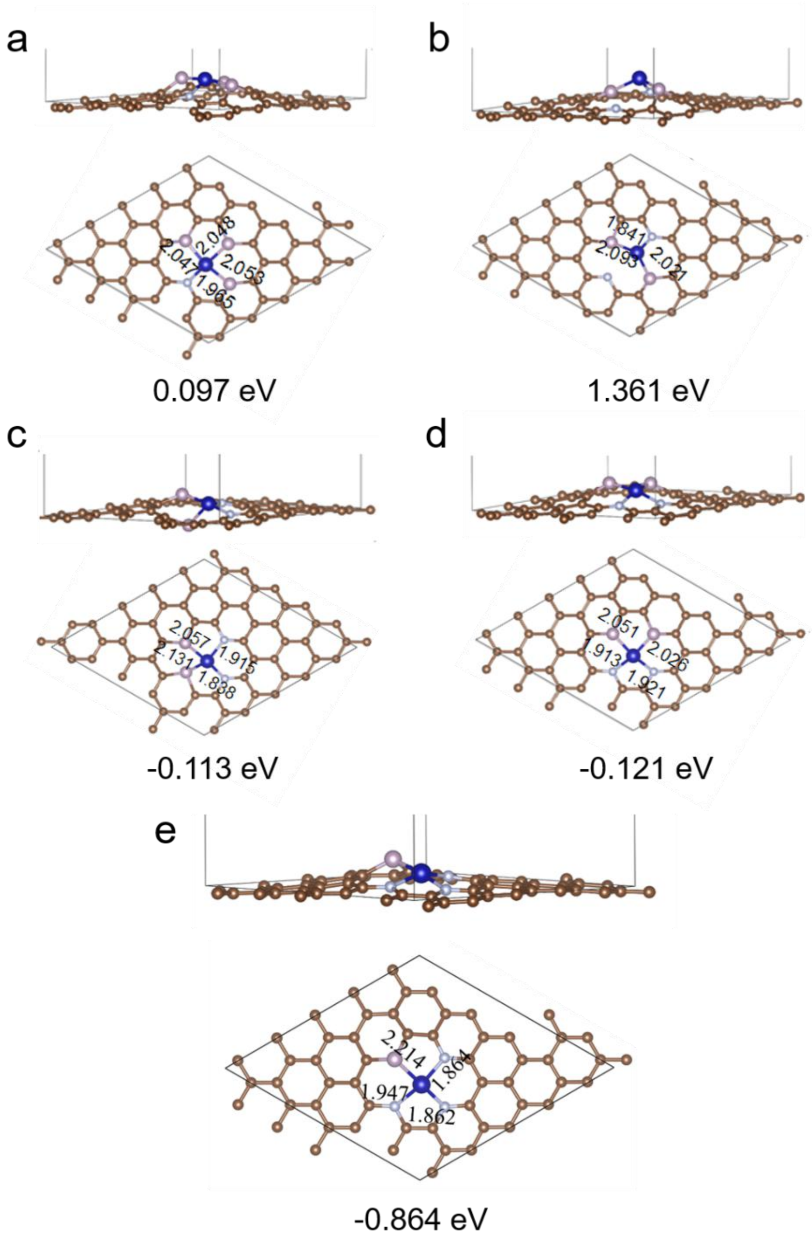

Supplementary Fig. 17 The side and top views and the formation energies of (a) Co_1-N_1P_3, (b) Co_1-N_1P_2, (c-d) Co_1-N_2P_2, and (e) Co_1-N_3P_1 structure.

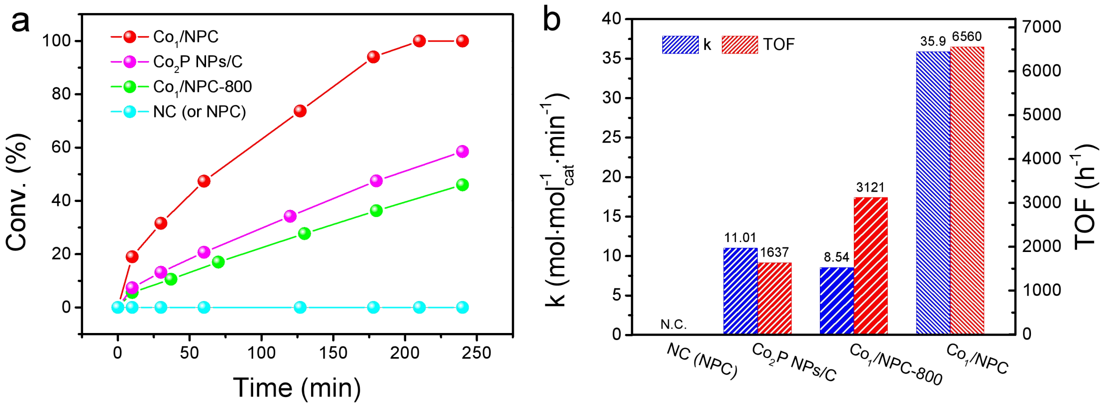

Supplementary Fig. 18 (a) Time course of nitrobenzene conversions over various samples. Reaction conditions: 5 mg catalyst, 2 mmol nitrobenzene, 40 mL EtOH/H_2O (v:v=4:1), 110 °C, 3 MPa H_2. (b) Reaction rates and TOF values of these samples for the nitrobenzene hydrogenation.

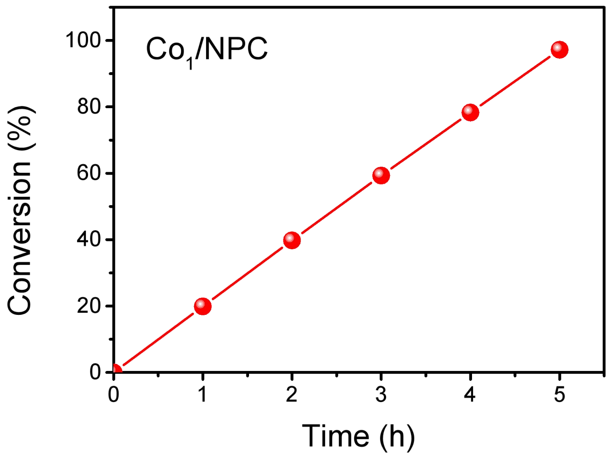

Supplementary Fig. 19 Time course of nitrobenzene conversions over Co$_1$/NPC sample. Reaction conditions: 22 mg catalyst, 0.5 mmol nitrobenzene, 40 mL EtOH/H_2O (v:v=4:1), 40 °C, 1 bar H_2.

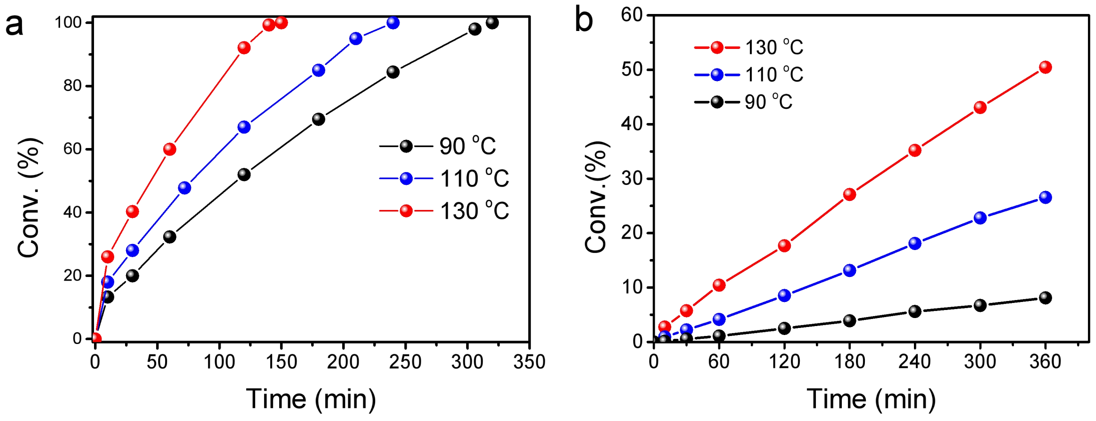

Supplementary Fig. 20 Hydrogenation conversions of nitrobenzene over (a) Co_1/NPC and (b) Co_1/NC under different temperatures. Reaction condition: 5 mg catalyst; 2 mmol nitrobenzene; 3 MPa H_2; 40 mL EtOH/H_2O (v:v=4:1).

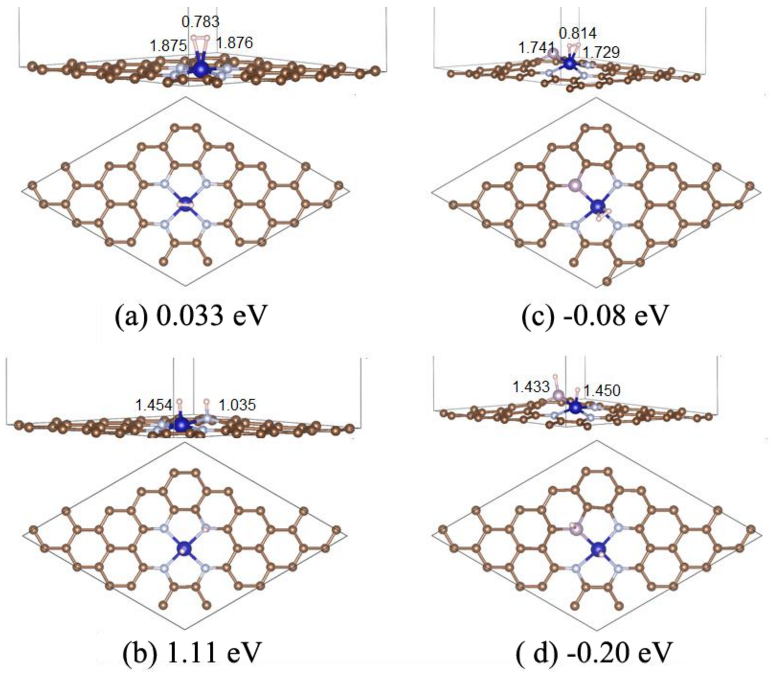

Supplementary Fig. 21 The initial states and final states for the direct dissociation of hydrogen over (a-b) Co_1/NC and (c-d) Co_1/NPC in the absence of water.

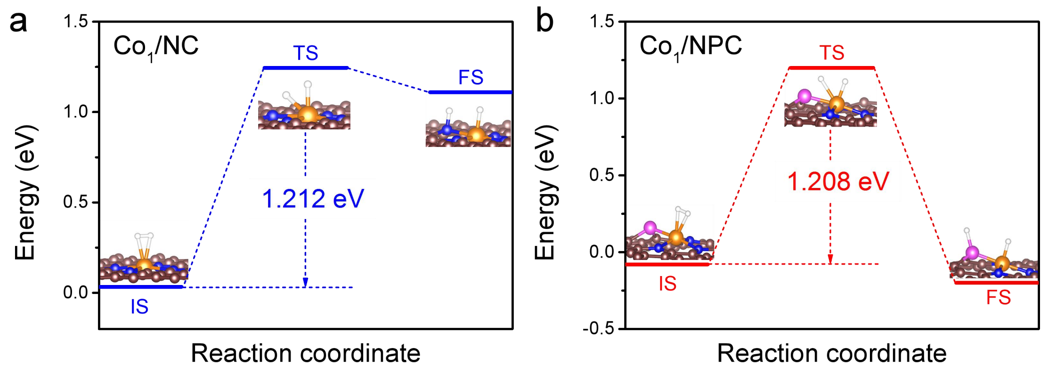

Supplementary Fig. 22 Energies profiles for the direct H_2 dissociation pathways over (a) Co_1/NC and (b) Co_1/NPC in the absence of water. IS, initial state; TS, transition state; FS, final state.

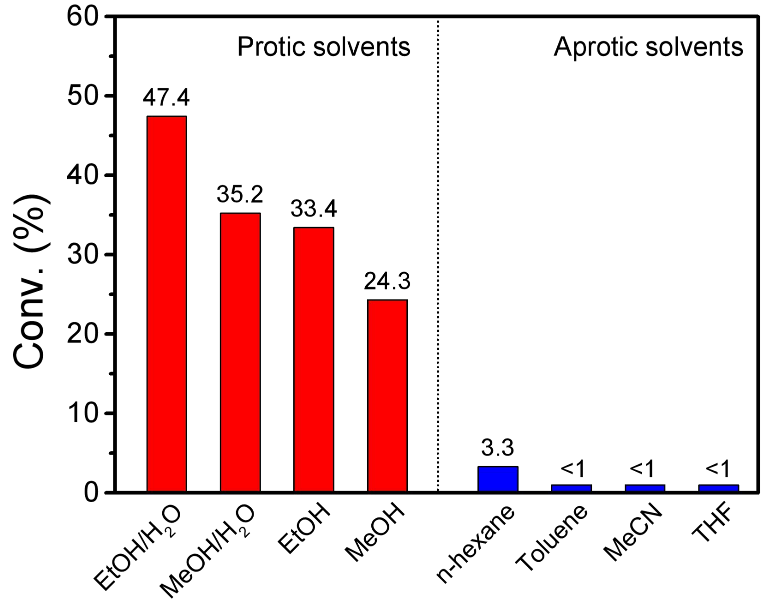

Supplementary Fig. 23 Catalytic performance of the hydrogenation of nitrobenzene over Co_1/NPC sample in different solvents. Reaction conditions: nitrobenzene, 2 mmol; various solvents, 10 mL; H_2, 3 MPa; temperature, 110 ^oC; reaction time, 1 h.

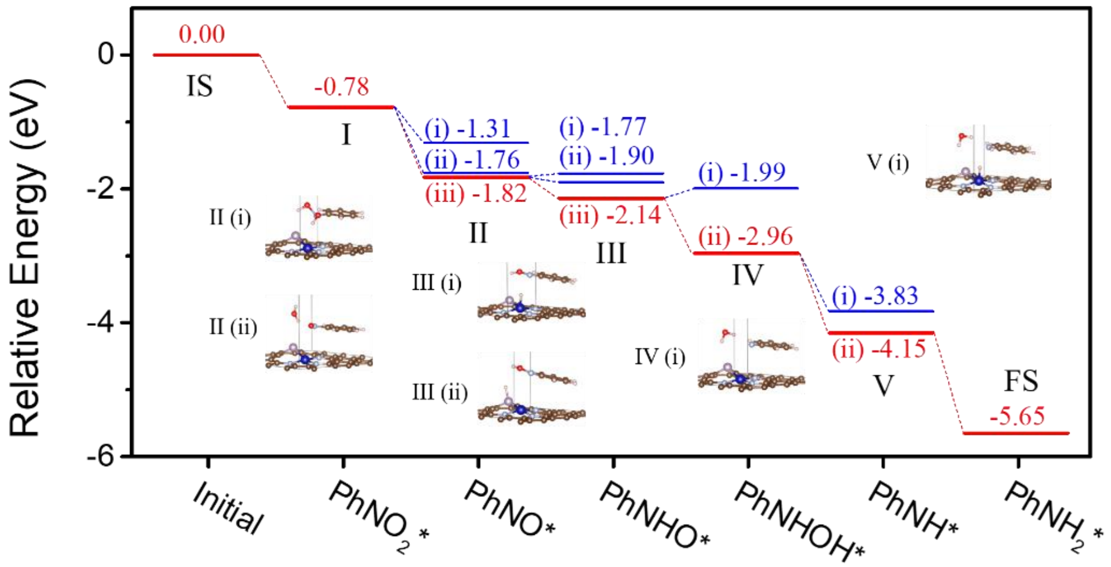

Supplementary Fig. 24 The optimization process of the reaction path of nitrobenzene hydrogenation over Co_1/NPC sample.

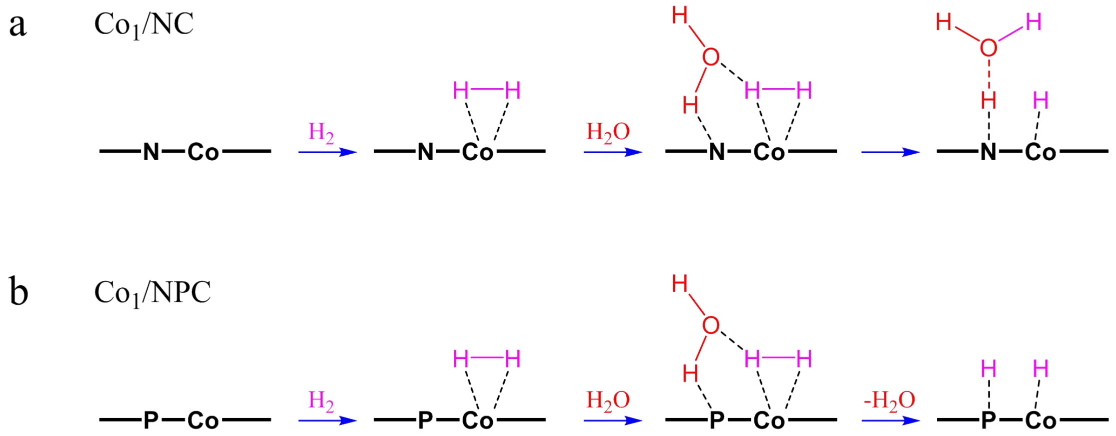

Supplementary Fig. 25 The pathways for the H_2O-mediated H-shuttling dissociation of hydrogen over (a) Co_1/NC and (b) Co_1/NPC.

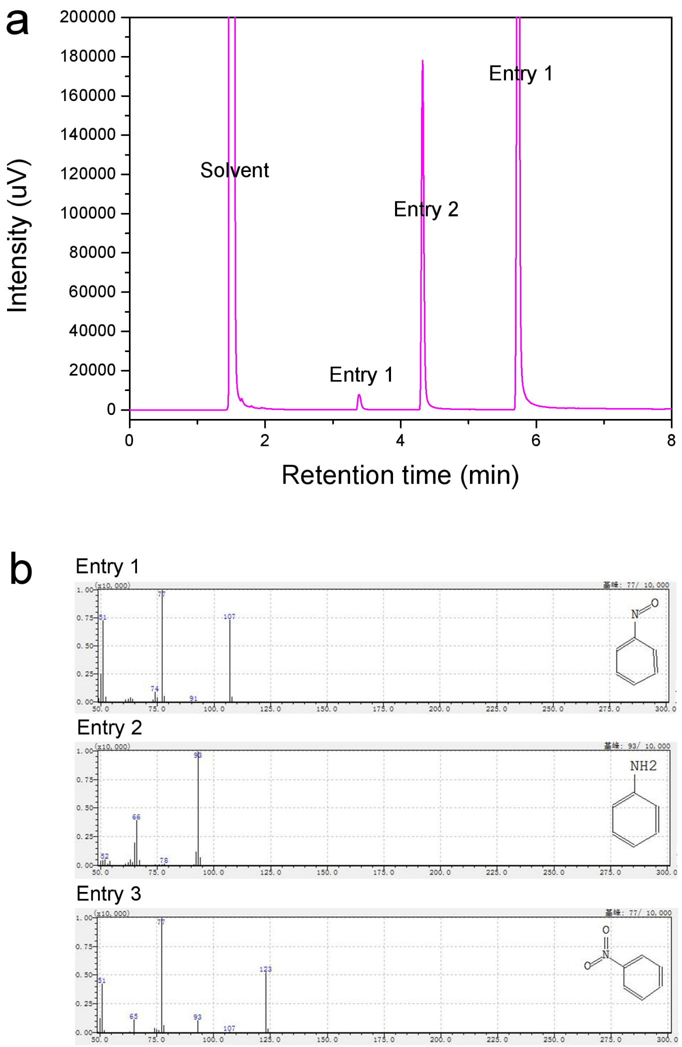

Supplementary Fig. 26 (a) GC of the mixture compositions of the reaction system within 10 min. (b) The mass spectrometric results of various compositions in (a).

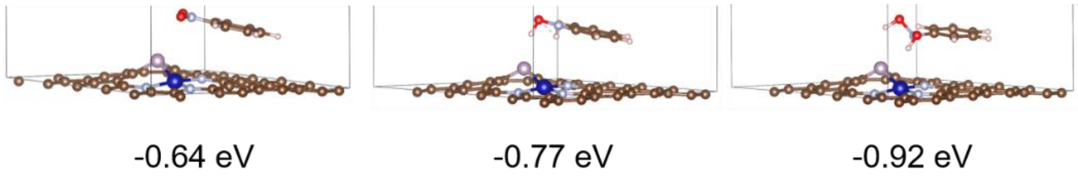

Supplementary Fig. 27 The calculated adsorption energies of Ph-NO_2, Ph-NHOH and Ph-NO on Co_1-N_3P_1 interface site.

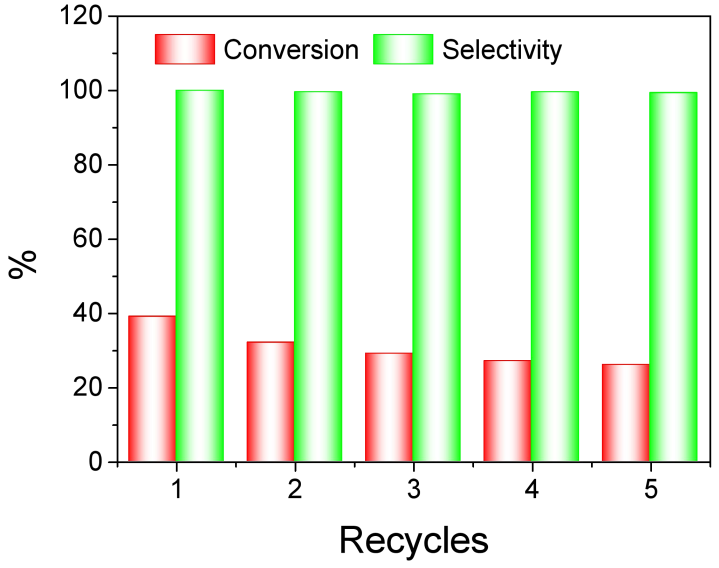

Supplementary Fig. 28 Stability test of Co_1/NPC for hydrogenation of nitrobenzene. Reaction conditions: 10 mg catalyst, 2 mmol nitrobenzene, 10 mL EtOH/H_2O (v:v=4:1), 110 °C, 3 MPa H_2, 10 min.

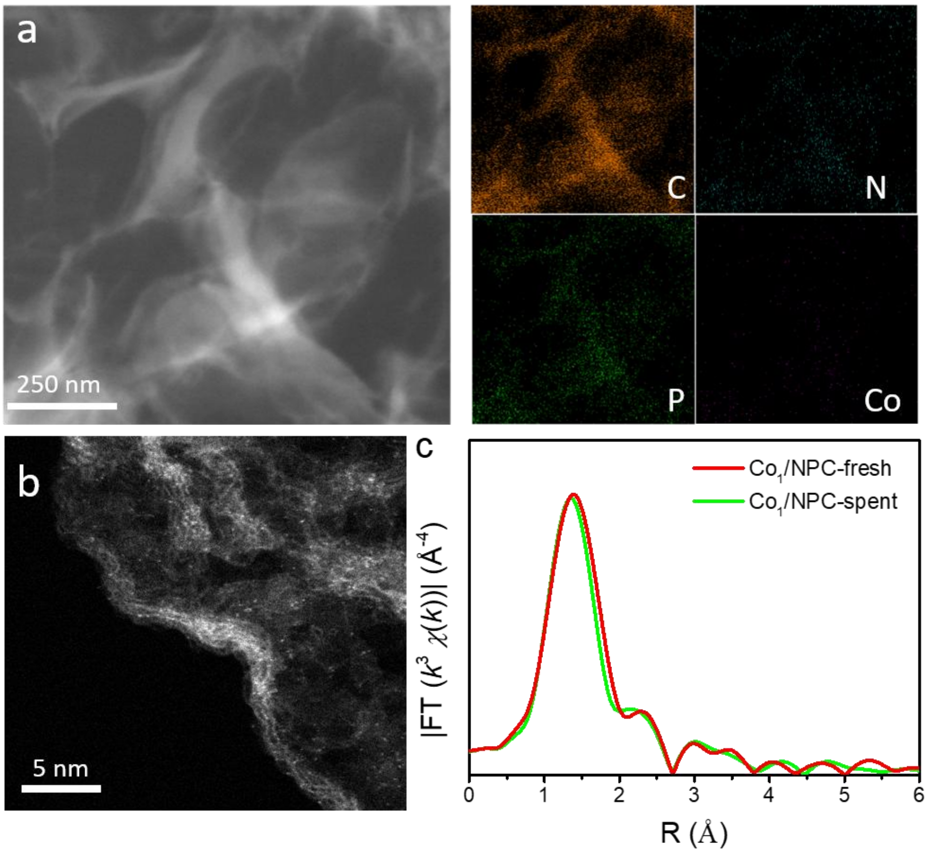

Supplementary Fig. 29 (a) HAADF-STEM image and corresponding EDS mapping distribution of Co_1/NPC-spent sample. (b) AC HAADF-STEM image and (c) Fourier transforms of the EXAFS spectra of Co_1/NPC-spent sample.

Supplementary Table 1. The Co contents of various samples estimated from ICP.

Samples	Co contents (wt%)
Co_1/NC	1.17
Co_1/NPC	0.45
Co_1/NPC-spent	0.42

Supplementary Table 2. EXAFS fitting parameters at the Co K-edge for various samples.

Sample	Shell	N [a]	R (Å) [b]	σ^2 (Å^2·10^{-3}) [c]	ΔE_0 (eV) [d]	*R* factor (%)
Co foil	Co-Co	12	2.49	6.3	6.3	0.084
Co_1/NC	Co-N	3.9	1.92	3.9	-8.7	0.6
Co_1/NPC	Co-N	3.2	1.93	2.7	-8.5	0.9
	Co-P	0.9	2.25	7.4		

[a] *N*: coordination numbers; [b] *R*: bond distance; [c] σ^2: Debye-Waller factors; [d] ΔE_0: the inner potential correction. *R* factor: goodness of fit. S_0^2 were set as 0.87 for Co-N, which was obtained from the experimental EXAFS fit of reference CoO by fixing CN as the known crystallographic value and was fixed to all the samples.

Supplementary Table 3. Comparison of catalysts for chemoselective hydrogenation of nitroarenes

Catalyst	Substrate	T (°C)	P (MPa)	t (h)	Substrate (mmol)	Con (%)	Sel. (%)	TOF (h^{-1})	Ref
Co_1/NPC	Nitrobenzene	110	3	3.5	2	100	100	5978	**This work**
Co_1/NPC	3-nitrostyrene	110	3	2	1	100	99.7	4499	**This work**
Co_1@NC-(SBA)	Nitrobenzene	90	1	1.5	0.5	99	99	22	ACS Catal. 2020, 10, 8672−8682
Co SAs/NC-800	Nitrobenzene	120	3	2.5	0.5	100	99.8	109.1	Adv. Funct. Mater. **2021**, 2103597
Ni–N–C-700	Nitrobenzene	120	3	10	0.25	99	99	8.4	Green Chem., 2019, 21, 704
Co–Nx/C-800-AT	Nitrobenzene	110	0.35	1.5	1	100	99	392	Sci. Adv. 2017;3: e1601945
Co SAs/NC	Nitrobenzene	110	3	4	3	99.7	99.1	76.8	ACS Appl. Mater. Interfaces 2020, 12, 34021−34031
Fe_1/N-C	Nitrobenzene	160	0.5	5	1	99	99	11	Sci. China Mater. 2021, 64, 642.
Co@mesoNC	Nitrobenzene	110	3	2	1	55	99	42	J. Catal. 357 (2018) 20–28
Co–N–C	Nitrobenzene	80	3	1.5	1	100	97	35.9	Chem. Sci., 2016, 7, 5758
Fe-P900-PCC	Nitrobenzene	100	4	18	1	99	99	0.5	Nat Commun. 2020, 11, 4074.
CoOx@NCNTs	Nitrobenzene	110	3	3	1	>99	98	-	ACS Catal. 2015, 5, 4783
Co SAs/NHPCN	Nitrobenzene	110	3	4.5	1	99	99	43.7	J. Phys. Chem. C 2021, 125, 5088
Co@NMC-800	Nitrobenzene	80	1	80 min	1	99	99	365	J. Catal. 348 (2017) 212
Co@NC	Nitrobenzene	110	3	3	1	36	>99	34	ChemCatChem 2017, 9,1854
Ni/C60	Nitrobenzene	110	2	5	20	79.6	82	230	Catal. Commun. 2017, 97, 83
Fe-phen/C-800	3-nitrostyrene	120	5	16	0.5	100	96	-	Science 342, 1073
Co-phen/C	3-nitrostyrene	110	5	6	0.5	99	92	8.4	Nature Chem 2013, 5, 537
Co-Phen	3-nitrostyrene	80	1	2	0.25	80.1	99.1	2.8	ACS Catal. 2021, 11, 3026
Co-Pc	3-nitrostyrene	80	1	2	0.25	80.3	97.8	9.6	ACS Catal. 2021, 11, 3026
Co@NMC-800	3-nitrostyrene	80	1	100 min	1	99	96	275	J. Catal. 348 (2017) 212
Co−Mo−S−0.39−180	3-nitrostyrene	150	1.1	7	0.25	>99	91	-	ACS Catal. 2017, 7, 2698
Co/C–N-600	3-nitrostyrene	100	10	1	0.5	99	96	5	J. Mol. Catal. A: Chem. 420 (2016) 56

Supplementary Table 4. Kinetic parameters of Co_1/NPC and Co_1/NC under different reaction temperatures.

Temperature (K)	k (s^{-1})	
	Co_1/NPC	Co_1/NC
363	0.2286	0.0092
383	0.3502	0.0295
403	0.4682	0.0539

The reaction rate constant (k) of the hydrogenation of nitrobenzene was calculated assuming a zero-order reaction as following:

$$C_x\text{-}C_0 = -kC_{cat}t \qquad (1)$$

where k is the zero-order rate constant (s^{-1}), t is the reaction time (s), C_x is the concentration of nitrobenzene at x=t time (mol/L), C_0 is the original concentration of nitrobenzene at x=0 s (mol/L), C_{cat} is the concentration of catalyst (mol/L).
